# Structural characterisation of hemagglutinin from seven Influenza A H1N1 strains reveal diversity in the C05 antibody recognition site

**DOI:** 10.1038/s41598-023-33529-w

**Published:** 2023-04-28

**Authors:** Seyed Mohammad Ghafoori, Gayle F. Petersen, Deborah G. Conrady, Brandy M. Calhoun, Matthew Z. Z. Stigliano, Ruth O. Baydo, Rena Grice, Jan Abendroth, Donald D. Lorimer, Thomas E. Edwards, Jade K. Forwood

**Affiliations:** 1 School of Dentistry and Medical Sciences, Charles Sturt University, Wagga Wagga, NSW 2650 Australia; 2 UCB BioSciences, Bainbridge Island, WA 98110 USA; 3 Seattle Structural Genomics Center for Infectious Disease (SSGCID), Seattle, WA 98109 USA

**Keywords:** X-ray crystallography, Virology

## Abstract

Influenza virus (IV) causes several outbreaks of the flu each year resulting in an economic burden to the healthcare system in the billions of dollars. Several influenza pandemics have occurred during the last century and estimated to have caused 100 million deaths. There are four genera of IV, A (IVA), B (IVB), C (IVC), and D (IVD), with IVA being the most virulent to the human population. Hemagglutinin (HA) is an IVA surface protein that allows the virus to attach to host cell receptors and enter the cell. Here we have characterised the high-resolution structures of seven IVA HAs, with one in complex with the anti-influenza head-binding antibody C05. Our analysis revealed conserved receptor binding residues in all structures, as seen in previously characterised IV HAs. Amino acid conservation is more prevalent on the stalk than the receptor binding domain (RBD; also called the head domain), allowing the virus to escape from antibodies targeting the RBD. The equivalent site of C05 antibody binding to A/Denver/57 HA appears hypervariable in the other H1N1 IV HAs. Modifications within this region appear to disrupt binding of the C05 antibody, as these HAs no longer bind the C05 antibody by analytical SEC. Our study brings new insights into the structural and functional recognition of IV HA proteins and can contribute to further development of anti-influenza vaccines.

## Introduction

Influenza virus (IV) is one of the most important viral pathogens impacting human health and the global economy^1^. It is estimated that around 10% of the global population is infected annually, with an economic burden of more than $87 billion (USD) on the healthcare system^2^. It is estimated that each year IV causes around 300–600 thousand deaths worldwide^3^. Humans have experienced several influenza pandemics in the last century. The most severe include Spanish (1918–1919), Asian (1957–1958), and Hong Kong (1968–1970) influenza, that cumulatively caused around 100 million deaths. Most recently, the 2009–2010 influenza pandemic (swine flu) resulted in 284,000 deaths^2,4^.

IV is a member of the *Orthomyxoviridae* family of segmented, negative-sense, single-stranded RNA viruses. There are four genera of IVs, A (IVA), B (IVB), C (IVC), and D (IVD). The genomes of IVA and IVB consist of eight RNA segments while the genomes of IVC and IVD consist of seven RNA segments, enveloped by a phospholipid bilayer derived from the host membrane. These segments encode for a variety of structural and non-structural proteins^5^. In IVA and IVB, two of these structural proteins, hemagglutinin (HA) and neuraminidase (NA), are inserted into the phospholipid bilayer as spikes^6^. HA is responsible for viral attachment, entry, and fusion into host cells, while NA cleaves the cell receptor to facilitate viral release^1^. In IVC and IVD, there is only a single spike protein, hemagglutinin-esterase-fusion (HEF), which is responsible for both viral attachment and release^7^.

In IVA there are 18 HA subtypes, phylogenetically divided into two groups. Mammalian IVA infection is initiated through HAs binding to α2,6 sialic acid-linkage galactose receptors on the host cell surface, before entering the cell via endocytosis^1^. Following endocytosis, HAs undergo conformational rearrangements due to the low pH of the endosome, leading to membrane fusion^8,9^. Influenza HA is synthesised as an immature precursor (HA0) that must be proteolytically cleaved into HA1 and HA2 polypeptides for the activation of membrane fusion, and a recent study characterized the structural differences of HA0 at neutral and low pH compared to HA1/HA2 at low pH^10^. Notably, the most commonly observed HA structure in the public database is the cleaved HA1/HA2 assembly at neutral pH. HA is a homotrimeric glycoprotein, with each monomer composed of two regions: (1) the head, comprising the receptor binding domain (RBD) which is crucial in viral attachment, and (2) the stalk, that is responsible for viral and cell membrane fusion in the endosome^11,12^. While the head contains a sialic acid binding pocket and plays the key antigenic role^12,13^, it has been shown that several antibodies can also recognise antigenic motifs on the stalk^14^.

The stalk is now being investigated for vaccine design as it is highly conserved in both IVA and IVB^15^. Constant antigenic drift causes a hypervariable area on the RBD, thus influenza vaccines targeting this area are inefficient. However, some anti-influenza antibodies, such as C05 and F045-092, have overcome this variation and can bind several IV strains within and across subtypes^14^. Some stalk-binding antibodies can also neutralise a wide range of IV strains. For instance, FI6v3 can neutralise all IVA strains and CR9114 can neutralise all IVA and IVB strains^14,16^. These conserved epitopes in the RBD and stalk look promising for designing a universal vaccine with higher effectiveness against circulating pathogenic IV strains. Recent advances in computational antigen design have generated new avenues for vaccine development against multiple IVA and IVB strains^17–19^.

Understanding HA structural variation across IVA and IVB strains is critical for antibody therapy and vaccine design, especially the hypervariability in the RBD which can lead to ineffective immune responses. As such, we characterised the crystal structures of HA from six H1 subtype IVA strains, with variations in the RBD. In addition, we determined the crystal structure of A/Denver/57 HA in complex with the anti-influenza antibody C05 fragment antigen binding (Fab). The structure and sequence of all seven HAs were then analysed to highlight similarities and differences. This study provides further insights into HA spike proteins from one of the most critical IV subtypes and expands our knowledge of HA structure and antibody binding.

## Materials and methods

### Cloning, expression, and purification of C05 Fab

C05 Fab was expressed and secreted from mammalian HEK293 cells (ThermoFisher; catalog R79007) in FreeStyle 293 media with PEI induction. The expressed protein construct contains a C-terminal non-cleavable His6 tag on the heavy chain. The protein was initially purified by nickel affinity chromatography on a HisTrap nickel Excel column in 20 mM Tris pH 8.0, 250 mM NaCl (Buffer A) supplemented with 500 mM imidazole Buffer B) with a 5–60% Buffer G linear gradient. The eluted protein was concentrated from ~ 0.1 to 1.26 mg/mL, then further purified by size exclusion chromatography on a Superdex 200 in 25 mM Tris pH 8.0, 150 mM NaCl. C05 Fab protein was then concentrated to 9.94 mg/mL and stored at − 80 °C prior to crystallisation.

### Cloning, expression, and purification of IVA HAs

Full length IVA H1N1 HAs were cloned from synthetic genes into vectors for expression in baculovirus infected insect cells (obtained from Expression Systems: website https://expressionsystems.com/products/cells) using a general expression and purification protocol described previously^20^. All IV open reading frames were cloned into a pBac vector with ampicillin resistance, encoding an N-terminal GP67 secretion sequence (sequence: MVLVNQSHQGFNKEHTSKMVSAIVLYVLLAAAAHSAFAGS) and a C-terminal thrombin-cleavable trimerisation domain followed by a His6 affinity tag (sequence: EFLVPRGSPGSGYIPEAPRDGQAYVRKDGEWVLLSTFLGHHHHHH). IVA HAs were expressed in baculovirus infected *T. ni* insect cells (obtained from Expression Systems: website https://expressionsystems.com/products/cells) (3% virus) using ESF-921 media and were harvested after 2 days. Media containing the secreted protein was buffer exchanged into 20 mM Tris pH 8.0, 150 mM NaCl and concentrated from 5 to 1 L using a Tangential Flow Filtration system. The protein was purified via nickel affinity chromatography on a Ni HiTrap chelating column. The eluted HA0 protein was treated with trypsin to generate mature HA (HA1 and HA2 polypeptides) and to remove the C-terminal trimerisation domain with His6 affinity tag. The protein was next purified by subtractive nickel affinity chromatography followed by size exclusion chromatography using a Superdex 200 column equilibrated with 25 mM Tris pH 8.0, 150 mM NaCl. The protein was concentrated to ~ 10 mg/mL and stored at − 80 °C prior to crystallisation.

The head of A/Fort Monmouth/1/1947 HA was cloned from a synthetic gene into a pET28a vector with kanamycin resistance, encoding an N-terminal thrombin-cleavable His6 tag (sequence: MGSSHHHHHHSSGLVPRG). The head domain was expressed in BL21(DE3) *E. coli* BL21(DE3) cells (from New England Biolabs, catalog number C2527H) in TB media overnight at 25 °C with 1 mM IPTG induction. Cells were harvested and lysed by sonication, then clarified in 250 mL of 50 mM Tris pH 8.5, 200 mM NaCl, 1 protease inhibitor tablet, 100 mg Lysozyme, 250 U Benzonase. Triton X-100 was added to 0.5% final concentration. The inclusion bodies were washed twice with 50 mM Tris pH 8.5, 200 mM NaCl, 10 mM EDTA, 10 mM beta mercaptoethanol, 1% Triton X-100, then twice with 50 mM Tris pH 8.5, 200 mM NaCl, 10 mM EDTA, 10 mM beta mercaptoethanol. The inclusion bodies were solubilised in 6 M guanidinium-HCl, 50 mM Tris pH 8.5, 150 mM NaCl, 10 mM beta mercaptoethanol. The protein was purified via nickel affinity chromatography on a HisTrap nickel Excel column and then refolded by dialysis over 2 days, initially with 50 mM Tris pH 8.5, 0.4 M l-arginine, 1 mM EDTA, 150 mM NaCl, 1 mM oxidized glutathione (GSSG), 1 mM reduced glutathione (GSH), then with 25 mM Tris pH 8.5, 150 mM NaCl. The His6 tag was removed by incubation with thrombin for 4 h on ice. The refolded protein was further purified by size exclusion chromatography on a Superdex 200 column in 50 mM Tris pH 8.5, 150 mM NaCl. The protein was concentrated to 5.04 mg/mL and stored at − 80 °C prior to crystallisation. Yield was 2.1 mg from 1.86 L of media.

### Complex formation analysis by analytical size exclusion chromatography

HA/C05 Fab complex formation was analysed by analytical scale size exclusion chromatography (anSEC). A Superose 6 analytical column was used on an Agilent 1100 HPLC with a running buffer of 25 mM Tris pH 8.5, 150 mM NaCl. Molecular weight standards were run before and after samples.

### Crystallisation and structure determination

IV HAs were crystallised at ~ 10 mg/mL using the sitting drop vapour diffusion method at 287 K in either Compact Jr or MRC crystallisation trays. Trays were set up either manually with 0.4/0.4 mL drop size or using a Formulatrix NT8 or TTP Mosquito drop setting robot with 0.2/0.2 mL drop size. Crystals were formed under the conditions provided in Supplementary Table 1. Crystals were harvested and flash‐frozen in liquid nitrogen. X-ray diffraction data were collected at 100 K the Advanced Light Source beamline 5.0.2 at 1.00 Å on a Dectris Pilatus3 S 6 M detector for A/Fort Monmouth/1/1947 trimer (7JPD) or Advanced Photon Source beamline 21 ID-G at 0.97856 Å for the A/Fort Monmouth/1/1947 refolded head domain (7JP4); all other X-ray data were collected at 100 K on a Rayonix MX‐300 mm CCD detector at a wavelength of 0.97872 Å on beamline 21‐ID‐F at Life Sciences Collaborative Access Team (LS‐CAT) at the Advanced Photon Source (APS, Argonne, IL). Data were reduced with the XDS/XSCALE package^21^. Structures were solved using molecular replacement using MorDA by using PDB ID 5VLI or other structures as a starting model as shown in Supplemental Fig. 3^22^. Models were built by iterative rounds of manual model building, and automated refinement was carried out using Coot^23^ and Phenix^24^. The quality of the structure was analysed using Molprobity^25^, and the final structure was deposited into the Protein Data Bank (PDB).

## Results

### Structural characterization of HA proteins from H1N1 IVA strains

The structural presentation of IV HA proteins are important determinants of disease severity and antibody recognition. Therefore, to better understand the structural details of HA proteins of H1N1 IVA strains and inform vaccination strategies, a total of 25 H1N1 IVA strains were nominated to the structure determination pipeline of the Seattle Structural Genomics Center for Infectious Disease^26^. Here we present the crystal structures of HAs from seven different H1N1 IVA strains (Supplementary Tables 2, 3). The full length mature HA1/HA2 trimer crystal structures are as follows: (1) A/Fort Monmouth/1/1947 solved at 2.95 Å resolution, (2) A/Jiangsu/ALS1/2011 solved at 2.35 Å resolution, (3) A/Almaty/32/1998 solved at 2.05 Å resolution, (4) A/Hickox/JY2/1940 solved at 1.95 Å resolution, (5) A/Netherlands/002P1/1951 solved at 2.5 Å resolution, and (6) A/Melbourne/1/1946 solved at 2.55 Å resolution. In addition, we also solved a 2.0 Å resolution crystal structure of the refolded head of A/Fort Monmouth/1/1947, which appears quite similar to the lower resolution full length mature trimer structure (Supplemental Fig. 3). Although we were unable to solve the structure of the A/Denver/57/1957 trimer in isolation, we solved a 2.92 Å resolution structure of this HA bound to the head-binding antibody C05 Fab.

**Figure 1:**
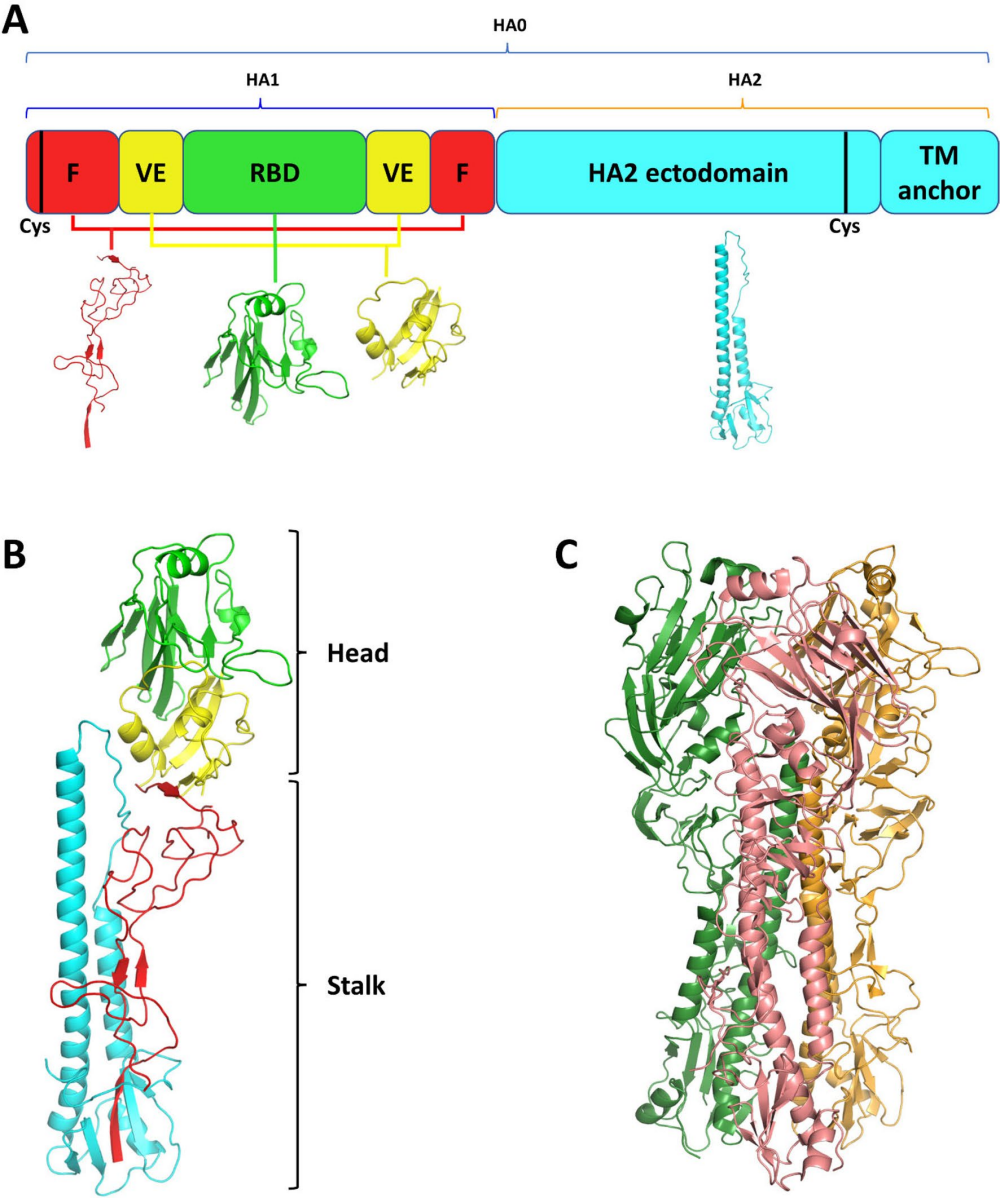
Influenza virus A hemagglutinin (HA) protein structure. (**A**) Schematic view of HA domains. *F* fusion domain (red), *VE* vestigial esterase domain (yellow), *RBD* receptor binding domain (green), *TM anchor* transmembrane anchor (cyan). The structure of each domain is shown under its schematic equivalent. Black lines depict cysteines that are responsible for disulfide bonds between HA1 and HA2. (**B**) Structure of A/Denver/57/1957 HA protomer (PDB ID: 6ML8), coloured by different domains. Colour coding as per panel (**A**). (**C**) Trimeric quaternary structure of HA, coloured by protomers.

A schematic overview of the various HA domains and their respective structures are shown in Fig. 1A,B, and here we use the structure of HA from A/Denver/57/1957 to illustrate the structural features. The immature, precursor form of HA (HA0) is 565 amino acids long and is cleaved into two main segments (HA1 and HA2) by trypsin-like proteases^27–31^. These HA1 and HA2 domains remain bound through an extensive network of interactions involving a disulfide bond between two conserved cysteines in the cleaved segments (Cys21 in the HA1 N-terminal domain and Cys480 in the HA2 C-terminal domain; Fig. 1A), in addition to 32 hydrogen bonds, 6 salt bridges, and a buried surface area of 2526 Å^2^ (Fig. 1A; Supplementary Fig. 2). HA1 has three main domains. The fusion domain (red) comprises N-terminal (1–59) and C-terminal (278–325) segments of HA1 that are bound together to form part of the stalk. The vestigial esterase (VE) domain (yellow) also comprises both N-terminal (60–112) and C-terminal (266–277) segments of HA1, but forms part of the head domain (Fig. 1A). The function of the VE domain in IVA and IVB is not well defined, however it does share 54% homology with the 9-*O*-acetylesterase domain in IVC (^32^) that cleaves the host receptor to facilitate viral budding^33^. The RBD (green) is located in the middle of HA1 (113–265) and is responsible for binding to sialic acid receptors, as well as mediating the release of viral ribonucleoprotein particles (vRNPs) into the cytoplasm^34,35^. HA2 is comprised of two main domains (cyan), including a long ectodomain (335–500) that is responsible for viral fusion and a C-terminal transmembrane (TM) anchor at the end. Collectively, the RBD and VE domain comprise the head, and the fusion domain, ectodomain, and TM anchor comprise the stalk of HA. Proteins, Interfaces, Structures and Assemblies (PISA)^36^ analysis predicted the biological assembly to be a trimer for all seven structures (Fig. 1C), however the number of interfacing residues was different between each (Supplementary Tables 4–10).

**Figure 2:**
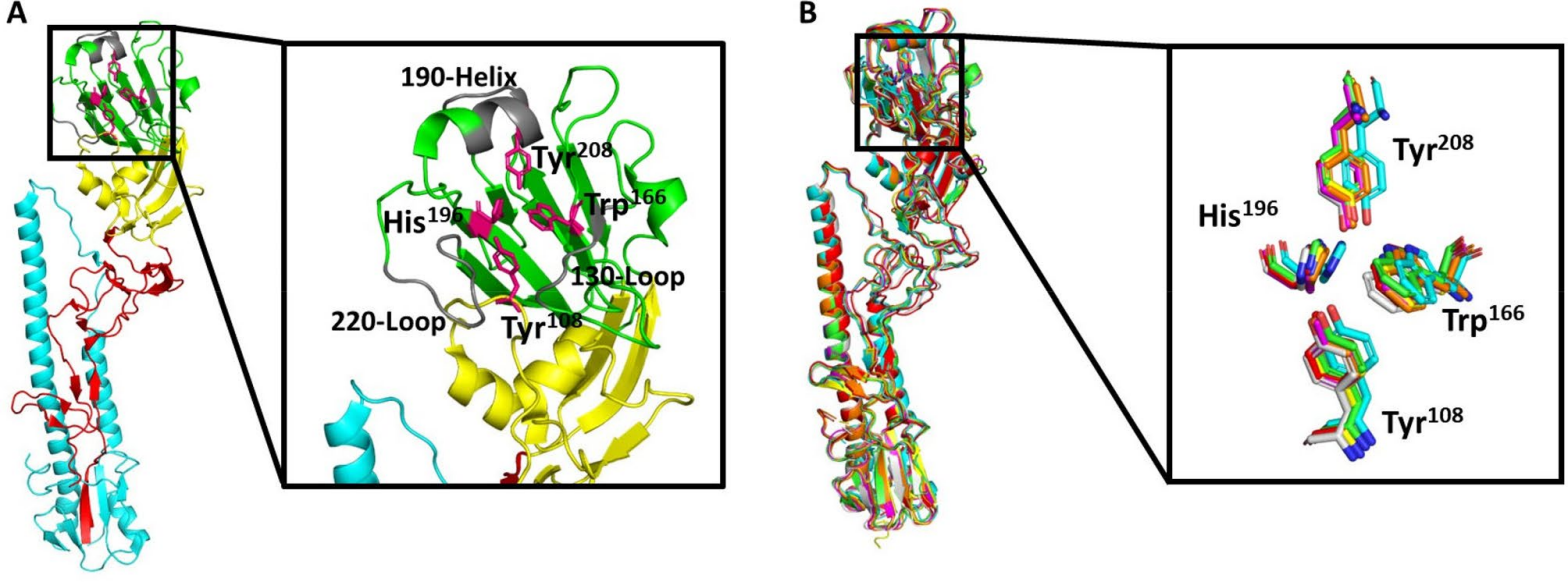
Sialic acid binding pocket within the receptor binding domain of influenza virus A (IVA) hemagglutinin (HA). (**A**) Structure of IVA HA (PDB ID: 6ML8), depicting the putative residues (dark pink)/features (grey) involved in sialic acid binding. Zoomed view to highlight the putative sialic acid binding pocket residues/features. (**B**) Structural alignment of IVA strains A/Denver/57 (PDB ID: 6ML8; green), A/Jiangsu/ALSI/2011 (PDB ID: 6D8W; cyan), A/Almaty/32/1998 (PDB ID: 6MYA; red), A/Netherlands/002P1/1951 (PDB ID: 6N41; yellow), A/Hickox/JY2/1940 (PDB ID: 6ONA; orange), A/Melbourne/1/1946 (PDB ID: 6OSR; light grey), A/Fort Monmouth/1/1947 (PDB ID: 7JPD; purple). Zoomed view to compare the positions of the putative sialic acid binding pocket residues.

The RBD is positioned in the membrane-distal region of all three monomers of the HA trimer. The sialic acid binding pocket within the RBD comprises two main components, the base, consisting of four highly conserved residues (Tyr^98^, Trp^153^, His^183^, Tyr^195^; Fig. 2A, shown in dark pink), and the sides, involving the 130-loop (135–138), 190-helix (190–198), and 220-loop (221–228) (Fig. 2A, shown in grey). Structural alignment shows that the base residues are conserved in all seven HA structures (Fig. 2B). Similarly, the sides are present in all seven HA structures, however there are fewer conserved residues, particularly in the 220-loop.

The stalk consists of the HA1 fusion domain, the HA2 ectodomain, and the TM anchor. The TM anchor is responsible for fusing to the endosomal membrane of the host cell^37^. HA1 and HA2 are linked via two Cys residues (Cys21 and Cys480) on the N-terminal of HA1 and C-terminal of HA2, as well as several hydrogen bonds (Supplementary Fig. 2).

**Figure 3:**
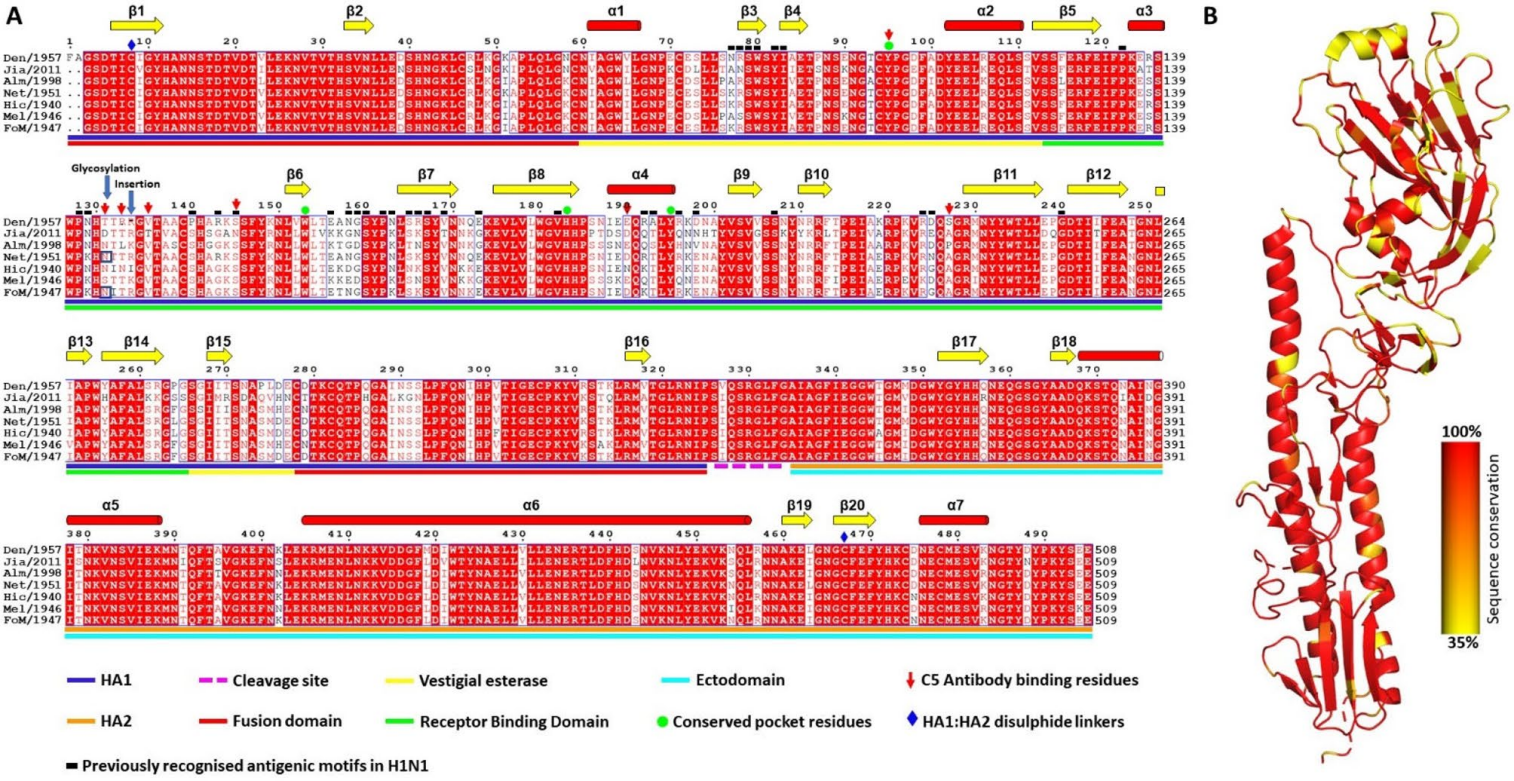
Sequence conservation among hemagglutinins (HAs) of seven influenza virus A (IVA) strains. (**A**) Multiple sequence alignment of HA from seven IVA strains. The secondary structural elements of the A/Denver/57 strain (PDB ID: 6ML8) are shown above the alignment. Conserved residues are highlighted in red. Residues of the HA1 segment and HA2 segment are shown with a blue and orange line below the alignment, respectively. The purple dashed line shows the cleavage site between the HA1 and HA2 segments. The second line below the alignment indicates the HA domains: red for the fusion domain, yellow for the vestigial esterase domain, green for the receptor binding domain, and cyan for the ectodomain. Putative sialic acid binding pocket residues are shown with green circles. Cysteines responsible for disulfide bonds between HA1 and HA2 segments are shown with blue diamonds. C05 binding residues on A/Denver/57 HA are indicated with red down arrows. Previously identified antigenic sites are indicated by black dashes. Multiple sequence alignment was performed using Clustal Omega^27^; the graphical output was generated by ESpript (https://espript.ibcp.fr/ESPript/ESPript/). (**B**) Structure of the IVA HA proteins coloured by sequence conservation, where red indicates 100% conservation and yellow indicates 35% sequence identity. Conservation was performed using PDB codes 6ML8, 6D8W, 6MYA, 6N41, 6ONA, 6OSR, and 7JPD in ConSurf server^40^.

HA sequence alignment of the seven IVA strains (Fig. 3A) reveals greater conservation (highlighted red) in the stalk than the head. Importantly, the four sialic acid binding pocket residues are completely conserved in all strains (Fig. 3A, green circles). As highlighted through a sequence conservation mapped onto the HA structure (Fig. 3B), the majority of non-conserved residues are within the RBD (Fig. 3A, green line), which has the potential to affect the efficacy of head-binding antibodies against different IV strains. Antigenic motifs in H1N1 IVA strains have been previously studied due to H1N1 IVA prevalence in the human population. Thirty two residues in five antigenic regions within the HA head^38,39^ are shown in Fig. 3 (black dashes), with only eleven of the residues conserved in all seven strains.

### Structural basis for the C05 interaction with the H1N1 influenza strain A/Denver/57

**Figure 4:**
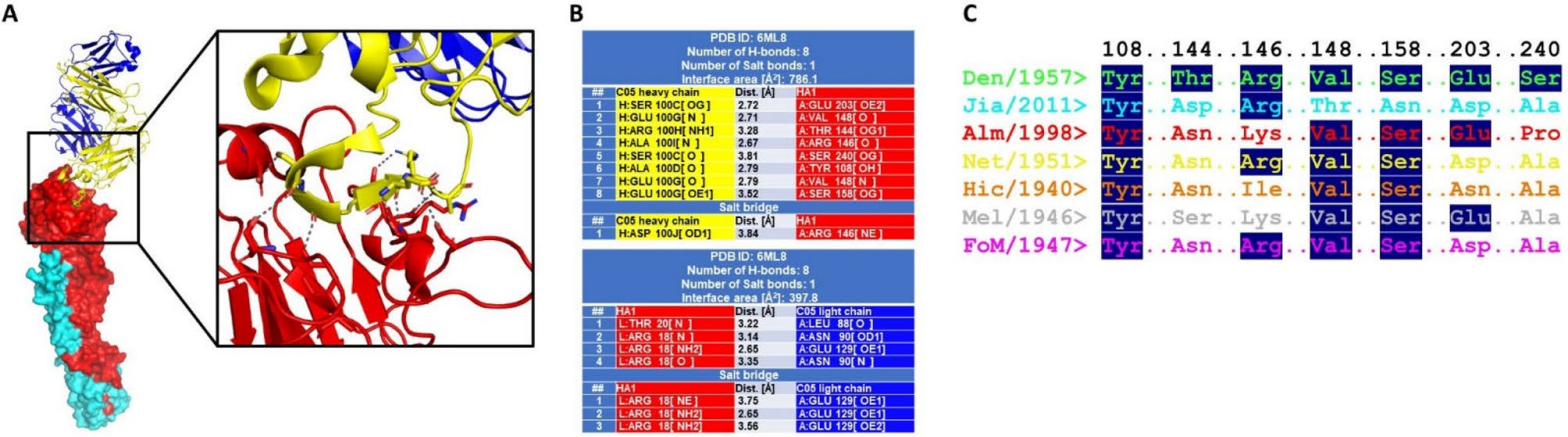
Analysis of the structure of HA from H1N1 influenza strain A/Denver/57 complexed with the antibody C05 Fab (PDB ID: 6ML8). (**A**) Structure of the HA: C05 complex. HA1 segment is in red; HA2 segment is in cyan; C05 heavy chain is in yellow; C05 light chain is in blue. Zoomed view to show the interaction between HA1 and the C05 heavy chain. Grey dashed lines indicate hydrogen bonds. (**B**) List of interactions between HA1 (red) and the C05 heavy chain (yellow) and light chain (blue). (**C**) Sequence alignment of the interacting residues in all seven HAs, compared to the HA: C05 complex (PDB ID: 6ML8).

Since there is no structure available of the C05 antibody bound to H1 (only H3^20,41,42^), to better understand how H1N1 IVA HA strains interact with the C05 antibody, we solved the crystal structure of HA from IV A/Denver/1957 bound to the head binding antibody C05 Fab at 2.92 Å resolution (PDB ID: 6ML8). Our structure showed a similar mode of antibody binding to that determined previously for C05 bound to HA from IV A/Hong Kong/1/1968^20^. PISA analysis of the HA:C05 complex revealed that seven HA residues mediate the interaction between the HA RBD antigenic motif and the C05 heavy chain, while only two HA residues mediate binding to the C05 light chain (Fig. 4A,B). Residues within the HA that mediate binding to the heavy chain include Tyr108, Thr144, Arg146, Val148, Ser158, Glu203, and Ser240, whilst HA residues interacting with the light chain include Arg18 and Thr20.

Comparing the structure of A/Denver/57 HA in complex with C05 Fab to our six other HA structures showed differences in the C05 antibody binding site (Fig. 4C). The only residue conserved in all seven structures is Tyr108, which is also one of the conserved sialic acid binding pocket sites. Thr144 is not conserved in any other structures and mutated to either Asp in A/Jiangsu/ALS1/2011 (PDB ID: 6D8W), Ser in the A/Melbourne/1/1946 strain, and Asn in the other strains. Arg146 was conserved with either a Lys or Arg in all strains except A/Hickox/JY2/1940, where an Ile residue was present. Val148 and Ser158 were both highly conserved, with one exception being A/Jiangsu/ALS1/2011, where these residues were Thr and Asn, respectively. Glu203 were conserved with negatively charged Asp/Glu residues with the exception of A/Hickox/JY2/1940, that contained an Asn. Finally, for Ser240, the most notable amino acid change was in A/Almatu/32/1998, which harboured a Pro at this position.

### Insertion between 146R-G147 influences C05 antibody binding

**Figure 5:**
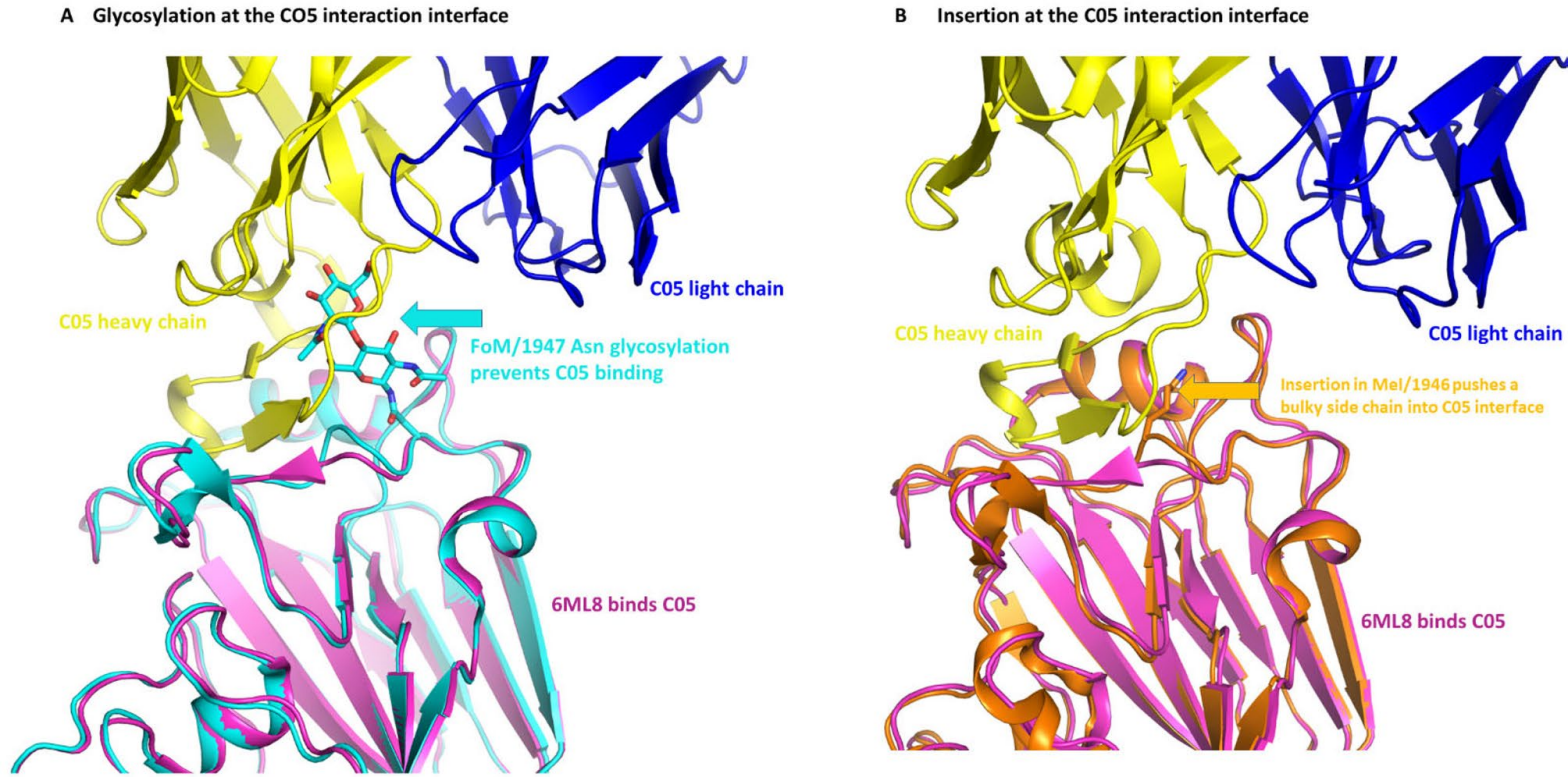
Structural analysis of the HA proteins from IVA H1N1 revealed two possible mechanisms for disruption at the C05 interface. (**A**) Potential mechanism 1 involved a glycosylation of Asn at NTTR of PDB 6N41 (see also Fig. 3A), and NITR of PDB 7JPD (see also Fig. 3A). (**B**) insertion of an amino acid between 146RG147 pushes a bulky residue side chain into the C05 binding interface. In both images, C05 heavy and light chain are coloured blue and yellow respectively, and 6ML8 HA is coloured magenta (note 6ML8 binds C05). In the left panel, 7JPD is coloured cyan (and does not bind C05 due to a glycosylation), and right panel, 6OSR, coloured orange is coloured orange, and has a loop insertion at the C05 interface.

The antibody C05 Fab failed to bind many of our other IVA HAs, as examined by analytical size exclusion chromatography (see data below). Failure to bind correlated with an insertion in HA between 146R-G147 of the epitope, whereas sequences without an insertion bound the antibody. For example, both A/Denver/57 (PDB ID: 6ML8, sequence: WPNHTTR/G147; Supplementary Fig. 4) and A/Iowa/1943 (sequence: WPKHTTG/G; Supplementary Fig. 6; structure not determined) lack an insertion at this site and show clear evidence of HA:C05 Fab complex formation. Examination of the HA alone structures provides insight into how certain sequences may evade recognition by the C05 antibody. Firstly, two structures, PDB 6N41 and 7JPD, contained glycosylated Asn residues at the Thr144 site (Figs. 3A, 4C), which could potentially physically block C05 antibody binding, while presumably retaining sialic acid binding (e.g. A/Netherlands/002P1/1951, PDB ID: 6N41, sequence: WPKHNTTR; and A/Fort Monmouth/1/1947, PDB ID: 7JPD, sequence: WPKHNITR); Fig. 5A. Secondly, the insertion positions a bulky residue (arginine, lysine, or isoleucine) into the C05 binding pocket (e.g. A/Jiangsu/ALSI/2011, PDB ID: 6D8W, sequence: WPNHDTT*R*; A/Almaty/32/1998, PDB ID: 6MYA, sequence: WPNHNTL*K*; A/Hickox/JY2/1940, PDB ID: 6ONA, sequence: WPNHNIN*I*; Supplementary Fig. 5; A/Melbourne/1/1946, PDB ID: 6OSR, sequence: WPKHSTT*K*; Supplementary Fig. 7) (Fig. 5B).

## Discussion

Antigenic drift, an evolutionary accumulation of amino acid substitutions in antigenic proteins determined by host adaptive immune systems^43^, is one of the key mechanisms used by viruses to avoid recognition by the host immune system. Antigenic drift can also increase viral attachment to its host receptor. For this to happen, viral surface proteins undergo high selective pressure during their evolution^44^. HA and NA are spike proteins found on the surface of IVA and IVB virions, with HAs the main target of the humoral immune system. In humans, there are two main categories of broadly neutralising antibodies (bnAbs) that target IV HAs, those that bind to either the head (specifically the RBD) or the stalk. Due to the high sequence conservation within the stalk, stalk-binding bnAbs such as FI6v3 and CR9114 can neutralise a wide range of IV HAs. Conversely, the neutralisation ability of head-binding bnAbs is much narrower due to the hypervariable sequence of the RBD^14^. C05 employs a unique approach, demonstrating a long complementarity-determining region (CDR) to bind the RBD, minimising contact with the hypervariable sequence^20^. This allows C05 to bind diverse HAs from H1, H2, H3 and H9 viruses^45^.

In the present study, we characterised the sequence and structure of HA from the H1N1 influenza strain A/Denver/57 bound to the bnAb C05 Fab and compared it to six additional H1N1 IVA HAs. Our analysis of all seven HA structures revealed that they adopt similar conformations and retain conserved putative sialic acid binding site residues, as seen in previously described IV HA structures. While further experiments are necessary to confirm the sialic acid binding site in our structures, highly conserved binding site residues in the previously characterised structure of IV HA with sialic acid suggests a similar binding site in our structures^12,34,46^.

The VE domain exists in all seven of our IVA HA structures, however its function in IVA and IVB is not well defined. In IVC, the similar 9-*O*-acetylesterase domain within HEF cleaves the sialic acid receptor on the host cell to release the virus and help viral budding, whereas NA is responsible for cell receptor cleavage in IVA and IVB^47^. Some studies show that the VE domain has antigenic properties, with several mouse (mAb) and human (hAb) antibodies, such as mAb H3v-47, mAb 1H5, mAb CR8071, mAb 100F4, mAb CR8071, hAb 46B8, and hAb 100F4, found to target this domain^48–52^. This suggests that the VE domain could be considered as a potential target for vaccine design, however the variability of this domain (as demonstrated in Fig. 3) may be an obstacle.

Recently, the HA stalk has become an interesting target for vaccine and drug design due to its conserved sequence and structure^16,34,53^. Within all seven of our IVA HA structures, the stalk is highly conserved, with reports that the stalk sequence is highly conserved both between and within IVA, IVB, and IVC. This allows some of the stalk-binding antibodies to bind and neutralise a variety of influenza strains^16^. hAbs CR6261 and F10 were found to neutralise most IVA group 1 strains by binding to the HA stalk^54,55^. Further, hAbs FI6v3 and CR9114 were found to bind IVA and both IVA and IVB, respectively^50,56^. These stalk-binding antibodies act by preventing conformational changes of HA, consequently hindering membrane fusion^50,54–56^. This highlights the potential of the HA stalk in the development of universal influenza vaccines.

C05 is a well-characterised human antibody that employs a long heavy chain CDR3 for binding to the RBD in the HA head. This method of binding minimises the area contacting the hypervariable residues in the RBD. Previous studies report that C05 binds H3 subtype HA through the antibody heavy chain, using a single long loop that allows it to decrease the contact area to ~ 550 Å^2^, considerably smaller than the usual antibody-antigen contact (~ 650 and ~ 740 buried surface area on HA by CH65 and 2D1 antibodies, respectively). C05 was claimed to bind H1 and H2 subtype HAs via a similar mechanism^20^. Interestingly, our structure of the H1N1 influenza strain A/Denver/57 HA bound to the bnAb C05 Fab indicates that both the heavy and light chains contribute to binding. Further, the surface area buried by the C05 heavy chain CDR is ~ 786 Å^2^, which is close to that of the 2D1 antibody. Our other IVA HA structures all contain an insertion near the C05 Fab CDR3 binding site that appears to prevent antibody binding, via either insertion of an Asn which becomes glycosylated to physically block C05 binding, or insertion of an Arg, Lys, or Ile into the C05 binding pocket. Interestingly, these insertions do not affect the identity or the positioning of the sialic acid binding residues, allowing these strains to bind the cell surface receptors while eliminating nearby potential antibody recognition sites. Moreover, this same loop-insertion position was identified in H3N2 HA domains that were responsible for loss of C05 binding^20^. A recent study assayed the capacity of C05 to bind diverse HA molecules (not examined here)^45^. Examination of those HA sequences revealed that insertions in the binding pocket could predict the ability of C05 to bind and recognise, underpinning the importance of this region in determining C05 specificity. Although the insertion of either a glycosylated residue observed in the electron density of our structures (A/Netherlands/002P1/1951 and A/Fort Monmouth/1/1947) or of a bulky side chain which partially changes the loop conformation (A/Jiangsu/ALSI/2011, A/Almaty/32/1998, A/Hickox/JY2/1940, and A/Melbourne/1/1946) appears consistent with the loss of C05 antibody Fab binding by analytical SEC, rigorous mutational analysis and quantitative binding experiments would be required to prove these are evasion mechanisms. It is possible, for example, that the glycosylation could be accommodated by movement of the positioning of the antibody as was the case for the S245N glycosylation site of H3N2 viral neuraminidases with IG01 antibodies (Ref PubMed ID 36543789).

## Supplementary Information

Supplementary Information.

## Data Availability

The datasets generated and analysed during the current study are available in the Protein Data Bank repository, https://www.rcsb.org. The PDBs codes are 6D8W, 6MYA, 6N41, 6ONA, 6OSR, 7JPD, 6ML8.
