# Supplementary material for: Structural characterisation of hemagglutinin from seven Influenza A H1N1 strains reveal diversity in the C05 antibody recognition site
Source: Sci Rep. 2023 Apr 28;13:6940. doi: 10.1038/s41598-023-33529-w (PMC10140725; doi:10.1038/s41598-023-33529-w)
Supplement: Supplementary file 1 — Supplementary Information. [file 41598_2023_33529_MOESM1_ESM.docx]

**Supplemental information**

Crystal structures of Influenza A virus hemagglutinins with variations in the receptor binding domains

Seyedmohammad Ghafoori et al.

**
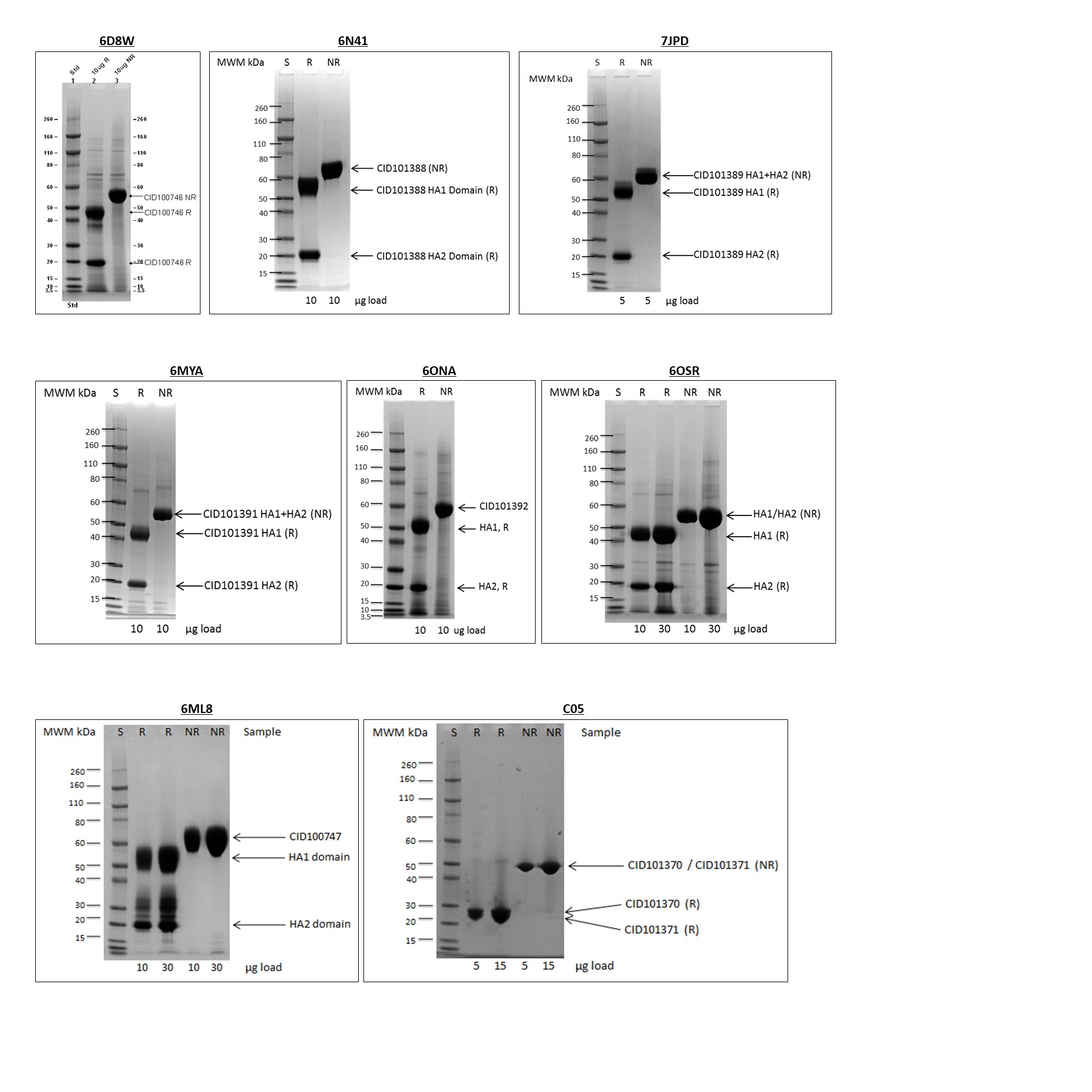
**

**
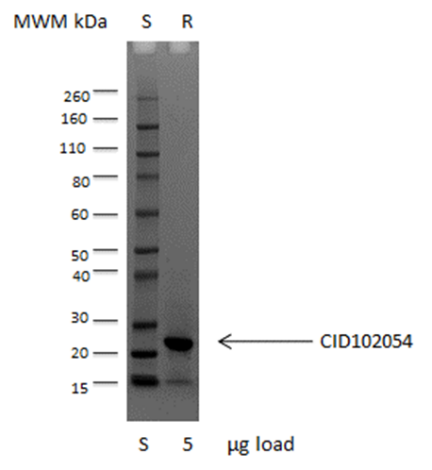
**

**7JP4 Refolded head domain A/Fort Monmouth/1/1947**

**Supplementary figure 1:** SDS PAGE analysis of HA and Fab samples


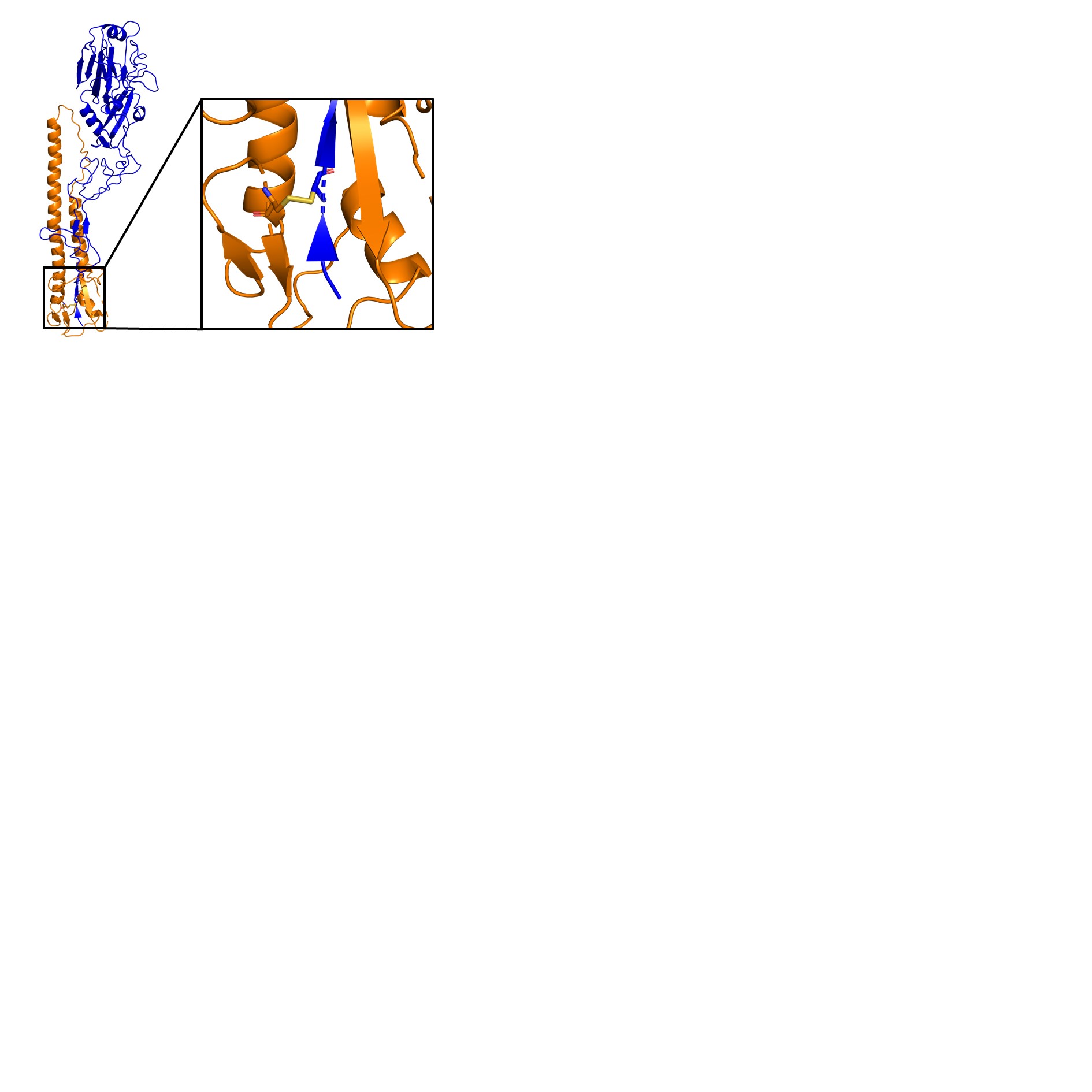


**Supplementary Figure 2**: Disulfate bond between HA1 and HA2 in A/Jiangsu/ALSI/2011 hemagglutinin (PDB ID: 6D8W)

**
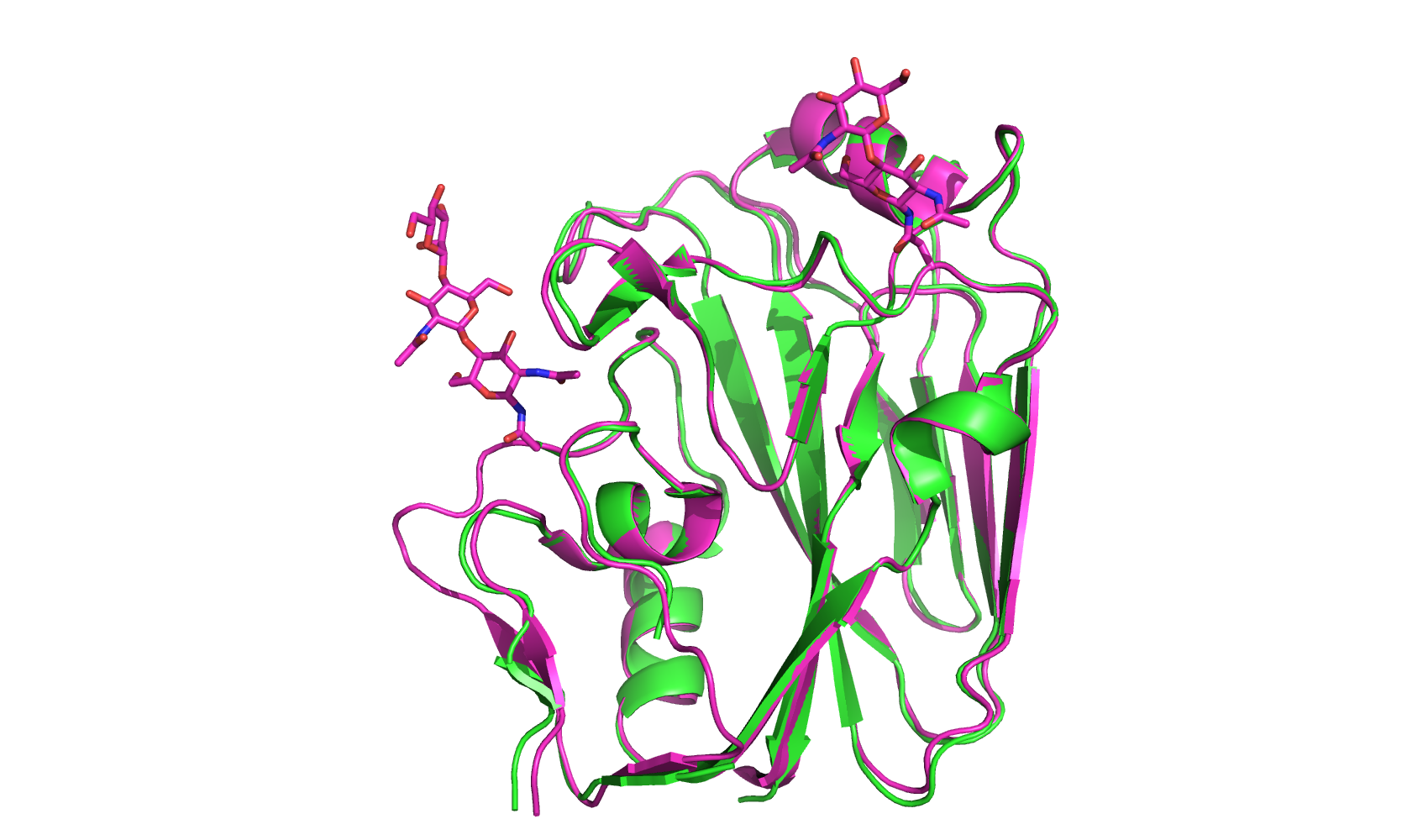
**

**Asn89**

**Asn129**

**Supplementary Figure 3**: Overlay of the crystal structures of insect cell expressed mature HA1/HA2 A/Fort Monmouth/1/1947 shown in magenta backbone with the glycans at Asn89 and Asn129 and the bacterial expressed and refolded HA head domain of A/Fort Monmouth/1/1947 shown in green backbone. For clarity and comparison purposes, only one head domain of the trimeric insect cell expressed HA structure is shown. Asn89 resides within the vestigial esterase (VE) domain, whereas Asn129 residues within the receptor binding domain (RBD).

**
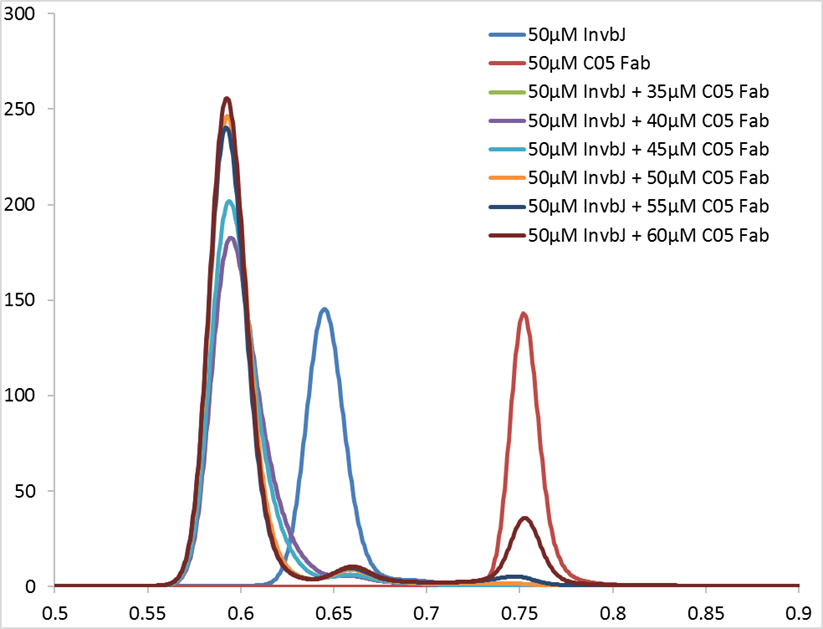
**

**Supplementary Figure 4**: Analytical SEC complex formation analysis for InvbJ.18715.a (A/Denver/57) HA with C05 antibody Fab. The HA alone appears at 0.65 column volumes (CV), the Fab alone appears at 0.75 CVs, and the HA/Fab complex appears at 0.59 CVs.

**
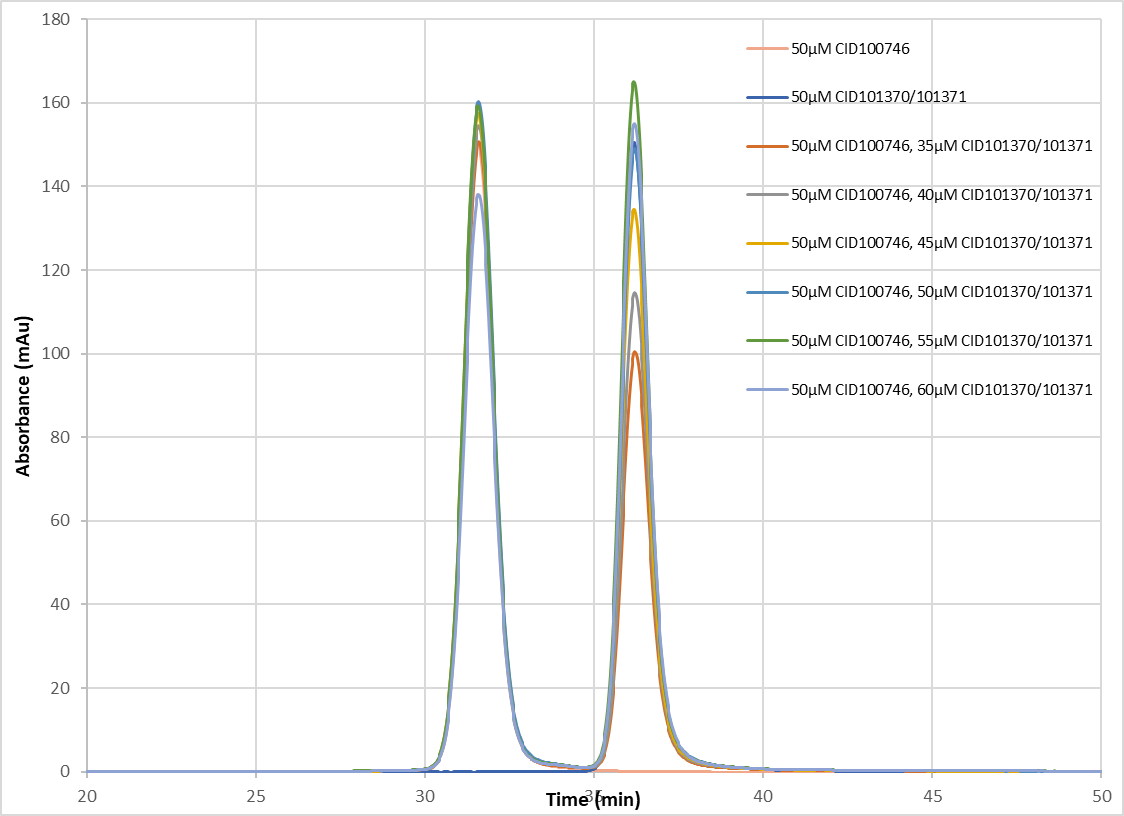
**

**Supplementary Figure 5**: Analytical SEC complex formation analysis for InvbI.18715.a (A/Jiangsu/ALSI/2011) HA with C05 antibody Fab. The HA alone appears at 32 minutes retention time, and the C05 Fab alone appears at 37 minutes retention time. The addition of the two samples does not result in HA/Fab complex formation at a new retention time.

**
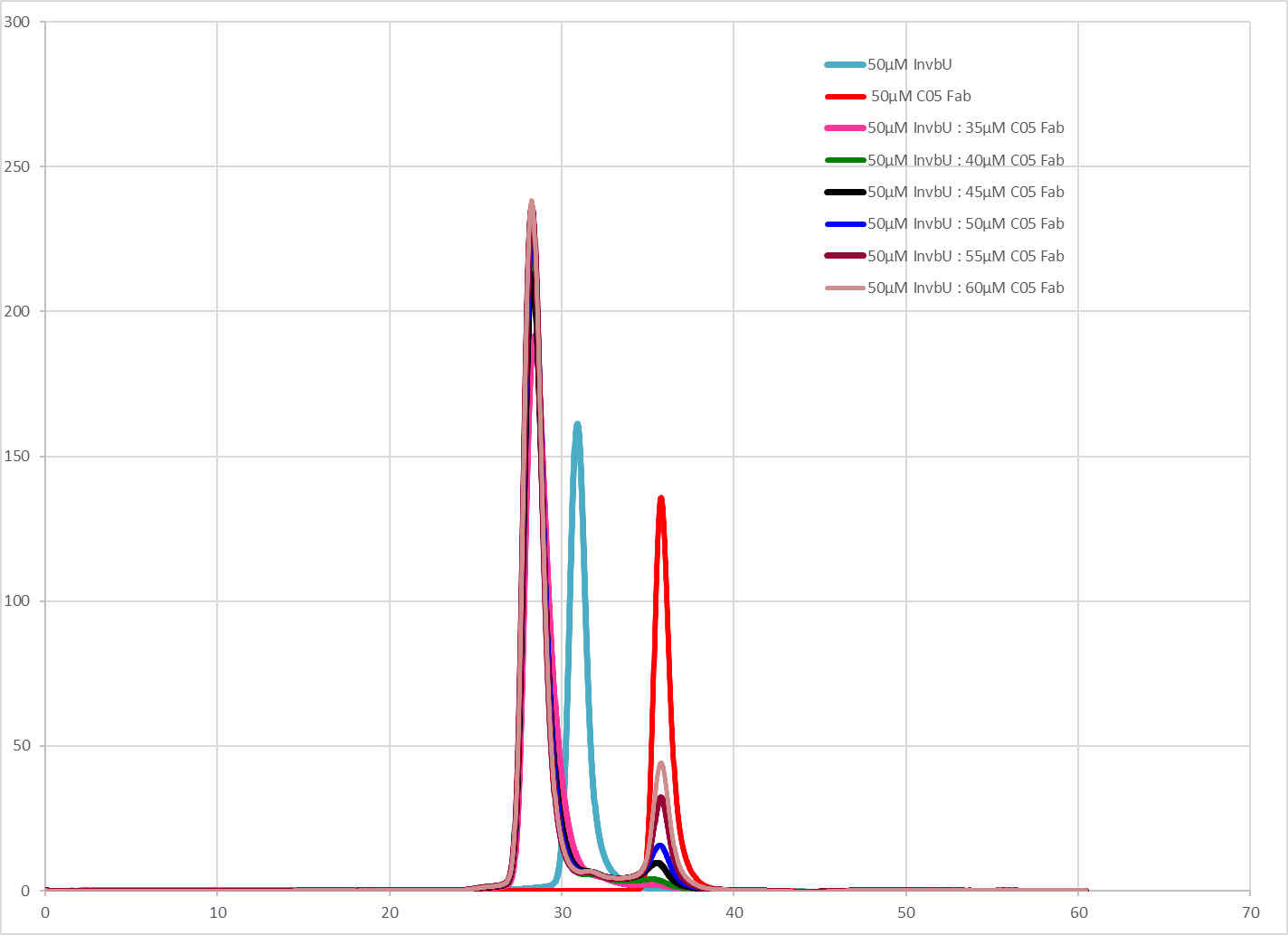
**

**Supplementary Figure 6**: Analytical SEC complex formation analysis for InvbU.18715.a (A/Iowa/1943) HA with C05 antibody Fab. The HA alone appears at 32 minutes retention time, the C05 Fab alone appears at 37 minutes retention time, and the complex appears at 28 minutes retention time.

**
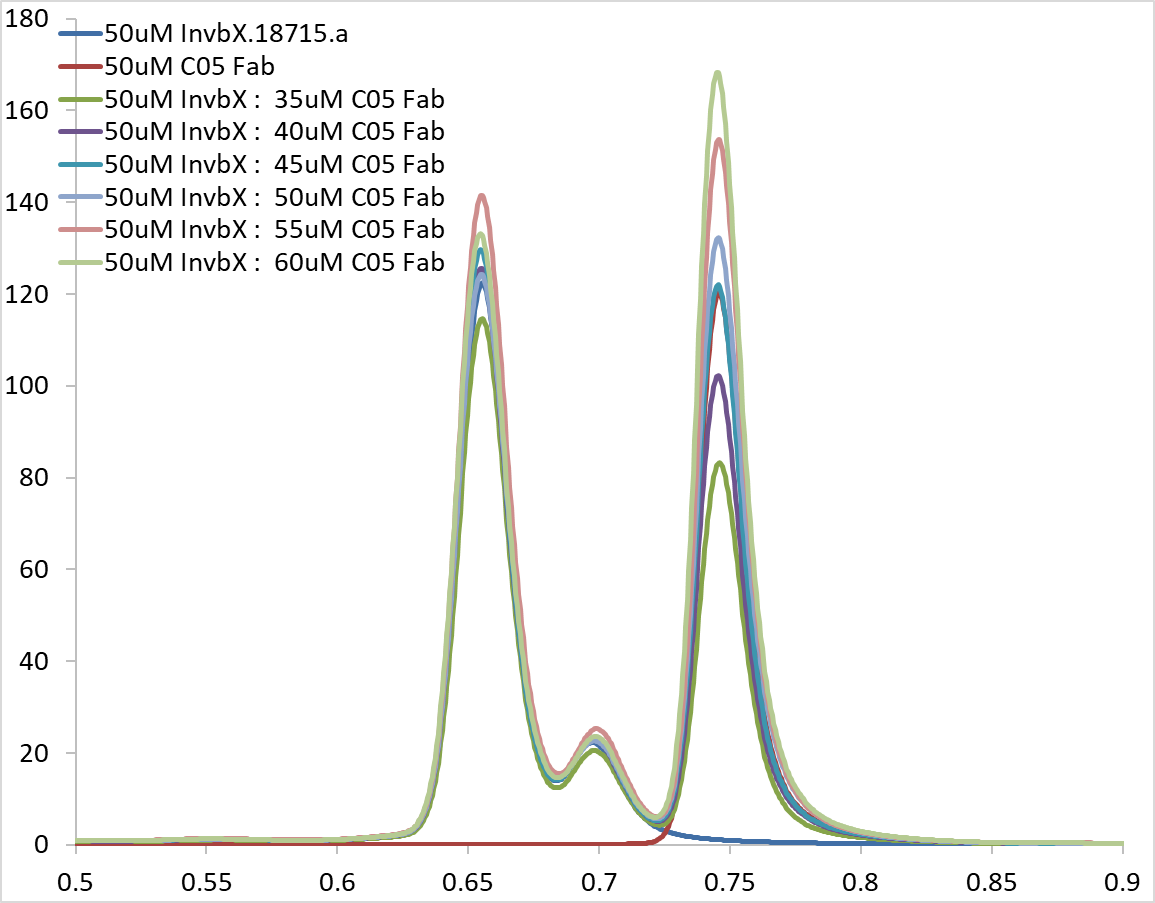
**

**Supplementary Figure 7**: Analytical SEC complex formation analysis for InvbX.18715.a (A/Melbourne/1/1946) HA with C05 antibody Fab. The HA alone appears at 0.66 CVs with an impurity at 0.70 CVs, and the C05 Fab alone appears at 0.75 CVs. Addition of the two components does not result in HA/Fab complex formation at a new retention time.

**Supplementary Table 1: Summary of crystallisation conditions for HA strains**

| **Protein** | **Concentration** | **Sample details** | **Screening condition** | **Crystallisation condition** | **cryo-protectant** |
| --- | --- | --- | --- | --- | --- |
| Full length mature HA A/Fort Monmouth/1/1947 | 9.84 mg/mL | SSGCID target ID InvbN.19815.a.KN11 SSGCID batch number PD38312 | MCSG1 | 0.2 M sodium iodide, 20% PEG 3350 | reservoir supplemented with 20% ethylene glycol |
| Refolded HA head domain A/Fort Monmouth/1/1947 | 5.04 mg/mL | InvbN.18715.a.TK11.PD38383 | Morpheus screen condition | 10% PEG 20,000, 20% PEG 550 MME, 0.1 M MOPS/Hepes pH 7.5, 20 mM glutamate, 20 mM alanine, 20 mM glycine, 20 mM lysine, 20 mM serine | reservoir supplemented with 20% ethylene glycol |
| Full length mature HA A/Jiangsu/ALS1/2011 | 10.18 mg/mL | InvbI.18715.a.KN11.PD38282 | Morpheus screen condition | 8.6% PEG 20,000, 17.2% PEG 500 MME, 0.1 M Hepes pH 6.8, 20 mM sodium formate, 20 mM ammonium acetate, 20 mM sodium citrate, 20 mM sodium potassium tartrate, and 20 mM sodium oxamate | reservoir supplemented with 20% ethylene glycol |
| Full length mature HA A/Almaty/32/1998 | 10.0 mg/mL | InvbP.18715.a.KN11.PD38330 | - | 15% PEG 6,000, 5% MPD, 0.1 M MES pH 6.5 | reservoir supplemented with 20% ethylene glycol |
| Full length mature HA A/Hickox/JY2/1940 | 9.5 mg/mL | InvbQ.18715.aKN11.PD38349 | - | 34% PEG 400, 0.1 M sodium citrate pH 5.5, 0.1 M magnesium chloride | reservoir supplemented with 20% ethylene glycol |
| Full length mature HA A/Netherlands/002P1/1951 | 10.4 mg/mL | InvbM.18715.a.KN11.PD38309 | - | 27.5% PEG 1500, 0.1 M MMT buffer (malic acid, MES, TRIS) pH 9 | reservoir supplemented with 15% ethylene glycol |
| Full length mature HA A/Melbourne/1/1946 | 10.1 mg/mL | InvbX.18715.a.KN11.PD38345 | - | 20% PEG 3350, 0.2 M potassium citrate | reservoir supplemented with 20% ethylene glycol |
| Full length mature HA A/Denver/1957 in complex with C05 Fab | 10 mg/mL | InvbJ.18715.a.KN11.PD38296/C05 Fab complex | - | 20% PEG 3350, 0,1 M BisTris Propane pH 7.5, 0.2 M potassium, sodium tartrate | reservoir supplemented with 20% ethylene glycol |

**Supplementary Table 2: Summary of structural deposition codes for various HA strains this study.**

| PDB ID | Influenza virus type | Place virus isolated | Stain number | Year of isolation | Subtype | Attached antibody |
| --- | --- | --- | --- | --- | --- | --- |
| 6D8W | A | Jiangsu | ALSI | 2011 | H1N1 | N/A |
| 6MYA | A | Almaty | 32 | 1998 | H1N1 | N/A |
| 6N41 | A | Netherlands | 002P1 | 1951 | H1N1 | N/A |
| 6ONA | A | Hickox | JY2 | 1940 | H1N1 | N/A |
| 6OSR | A | Melbourne | 1 | 1946 | H1N1 | N/A |
| 7JPD | A | Fort Monmouth | 1 | 1947 | H1N1 | N/A |
| 6ML8 | A | Denver | 57 | 1957 | H1N1 | C05 |

**Supplementary Table 3: Summary of crystallographic statistics for data collection and refinement.**

| **Data collection and processing** | **A/Jiangsu/ALSI/2011**  **(H1N1)** | **A/Almaty/32/1998**  **(H1N1)** | **A/Netherlands/002P1**  **/1951**  **(H1N1)** | **A/Hickox/JY2/1940**  **(H1N1)** | **A/Melbourne/1/1946 (H1N1)** | **A/Fort Monmouth/1/1947**  **(H1N1)** | **A virus A/Denver/57**  **(H1N1)** |
| --- | --- | --- | --- | --- | --- | --- | --- |
| PDB accession code | 6D8W | 6MYA | 6N41 | 6ONA | 6OSR | 7JPD | 6ML8 |
| MR model | 5VLI | 6D8W | 6MYA | 6MYA | 6MYA | 5VLI | 4EDB, 4FNL |
| SSGCID Target ID | InvbI.18715.a | InvbP.18715.a | InvbM.18715.a | InvbQ.18715.a | InvbX.18715.a | InvbN.18715.a | InvbJ.18715.a |
| Construct ID | CID100746 | CID101391 | CID101388 | CID101392 | CID101399 | CID101389 | CID100747 |
| Wavelength (Å) | 0.97872 | 0.97872 | 0.97872 | 0.97872 | 0.97872 | 1.00 | 0.97872 |
| Resolution range (Å) | 43.292-2.35 (2.41-2.35) | 45.928-2.05 (2.1-2.05) | 46.274-2.5 (2.56-2.5) | 45.388-1.95 (2.0-1.95) | 48.736-2.55 (2.62-2.55) | 48.85-2.95 (3.03-2.95) | 47.886-2.92 (3.0-2.92) |
| Space group | *P*1 | *P*2_1_ | *P*2_1_ | *C*2 | *P*2_1_ | *P*2_1_2_1_2_1_ | *P*321 |
| Unit cell (Å, °) | 67.3, 113.33, 130.12, 110.16, 90.85, 90.28 | 69.65, 244.37, 115.02, 90, 90.09, 90 | 70.28, 123.41, 119.97, 90, 95.54, 90 | 108.56, 102.25, 160.95, 90, 91.07, 90 | 73.1, 113.97, 246.38, 90, 91.44, 90 | 131.79, 142.36, 234.23, 90, 90, 90 | 118.46, 118.46, 162.44, 90, 90, 120 |
| Unique reflections | 146,567 (10,858) | 231,107 (17,159) | 70,379 (5165) | 127,783 (9450) | 131,565 (9715) | 91,660 (6805) | 29,212 (2117) |
| Multiplicity | 2.64 (2.66) | 3.28 (3.28) | 4.27 (4.29) | 5.18 (5.30) | 5.26 (5.28) | 6.26 (6.97) | 7.32 (7.56) |
| Completeness (%) | 97.4 (96.9) | 96.5 (97.2) | 99.9 (100) | 99.9 (99.9) | 99.8 (99.7) | 98.2 (100) | 99.9 (100) |
| Mean I/sigma(I) | 2.13 | 2.36 | 2.11 | 2.13 | 1.52 | 2.0 | 2.0 |
| Wilson B-factor Å2 | 40.16 | 37.812 | 50.901 | 38.193 | 46.927 | 57.51 | 66.752 |
| R-merge % | 0.063 (0.549) | 0.065 (0.545) | 0.092 (0.791) | 0.077 (0.875) | 0.168 (1.089) | 0.145 (0.828) | 0.107 (97.7) |
| **Refinement** | | | | | | | |
| Number of reflections | 146516 | 231055 | 70315 | 127689 | 131524 | 91560 | 29208 |
| Number of R-free reflections | 2041 | 2002 | 2032 | 2048 | 1954 | 4458 | 1534 |
| R-work % | 17.92 | 18.81 | 18.70 | 19.30 | 19.12 | 25.38 | 20.12 |
| R-free % | 22.47 | 22.18 | 23.50 | 22.97 | 24.59 | 28.64 | 26.42 |
| RMS (bonds) | 0.008 | 0.007 | 0.008 | 0.007 | 0.008 | 0.004 | 0.005 |
| RMS (angles) | 0.940 | 0.892 | 1.004 | 0.835 | 0.982 | 0.686 | 0.744 |
| **Ramachandran plot** | | | | | | | |
| Favored (%) | 96.66 | 97.67 | 97.42 | 97.48 | 96.89 | 97.28 | 92.49 |
| Allowed (%) | 99.82 | 99.86 | 100 | 99.93 | 100 | 99.83 | 99.34 |
| Outliers (%) | 0.18 | 0.14 | 0 | 0.07 | 0 | 0.17 | 0.66 |

**Supplementary Table 4: Comparison between three interfaces in A/Jiangsu/ALSI/2011 hemagglutinin**

| **PDB ID: 6D8W**  **Number of H-bonds: 15**  **Number of Salt bonds: 9**  **Interface area [Å2]: 1600.0** | | | **PDB ID: 6D8W**  **Number of H-bonds: 13**  **Number of Salt bonds: 8**  **Interface area [Å2]: 1438.7** | | | **PDB ID: 6D8W**  **Number of H-bonds: 17**  **Number of Salt bonds: 11**  **Interface area [Å2]: 1521.7** | | |
| --- | --- | --- | --- | --- | --- | --- | --- | --- |
| **Protomer 1** | [**Dist. [Å]**](javascript:openWindow('pi_ipage_atmdist.html',400,250);) | **Protomer 2** | **Protomer 3** | [**Dist. [Å]**](javascript:openWindow('pi_ipage_atmdist.html',400,250);) | **Protomer 2** | **Protomer 1** | [**Dist. [Å]**](javascript:openWindow('pi_ipage_atmdist.html',400,250);) | **Protomer 3** |
| F:ASN 379[ ND2] | 3.72 | A:THR  20[ O  ] | D:ARG 219[ NH2] | 3.77 | A:SER 205[ O  ] | F:ARG 219[ NH2] | 3.18 | D:SER 205[ O  ] |
| F:ASN 379[ ND2] | 3.25 | A:ILE  21[ O  ] | D:GLU 403[ N  ] | 3.49 | A:GLU 102[ OE1] | F:GLU 403[ N  ] | 3.29 | D:GLU 102[ OE1] |
| F:TYR 209[ OH ] | 3.41 | A:GLU 215[ OE2] | D:LYS 404[ N  ] | 2.85 | A:GLU 102[ OE1] | F:LYS 404[ N  ] | 2.75 | D:GLU 102[ OE1] |
| F:ASN 446[ ND2] | 2.68 | A:LEU 331[ O  ] | D:LYS 404[ NZ ] | 3.87 | A:GLN 106[ OE1] | F:ARG 405[ N  ] | 3.03 | D:GLU 102[ OE2] |
| F:SER 205[ O  ] | 3.49 | A:ARG 219[ NH2] | D:ARG 405[ N  ] | 3.04 | A:GLU 102[ OE2] | F:ARG 405[ NE ] | 2.62 | D:GLU 102[ OE2] |
| F:GLU 102[ OE1] | 3.41 | A:GLU 403[ N  ] | D:ARG 405[ NE ] | 2.75 | A:GLU 102[ OE2] | F:ARG 405[ NH1] | 2.98 | D:GLU 398[ O  ] |
| F:GLU 102[ OE1] | 2.88 | A:LYS 404[ N  ] | D:ARG 405[ NH1] | 2.82 | A:GLU 398[ O  ] | F:ARG 405[ NH1] | 2.94 | D:GLU 403[ OE1] |
| F:GLU 102[ OE2] | 3.23 | A:ARG 405[ N  ] | D:ARG 405[ NH1] | 2.81 | A:GLU 403[ OE1] | F:ARG 405[ NH2] | 3.01 | D:GLU 403[ OE2] |
| F:GLU 102[ OE2] | 2.70 | A:ARG 405[ NE ] | D:ARG 405[ NH2] | 2.59 | A:GLU 403[ OE2] | F:ASN 408[ ND2] | 2.75 | D:GLU 105[ OE2] |
| F:GLU 398[ O  ] | 3.05 | A:ARG 405[ NH1] | D:ASN 408[ ND2] | 2.79 | A:GLU 105[ OE1] | F:LYS 412[ NZ ] | 2.94 | D:ASN 410[ OD1] |
| F:GLU 403[ OE1] | 2.83 | A:ARG 405[ NH1] | D:LYS 412[ NZ ] | 2.93 | A:ASN 410[ OD1] | F:LYS 412[ NZ ] | 2.55 | D:ASP 414[ OD1] |
| F:GLU 403[ OE2] | 2.79 | A:ARG 405[ NH2] | D:LYS 412[ NZ ] | 2.52 | A:ASP 414[ OD1] | F:THR  20[ O  ] | 3.58 | D:ASN 379[ ND2] |
| F:GLU 105[ OE2] | 2.76 | A:ASN 408[ ND2] | D:GLU 215[ OE2] | 3.78 | A:TYR 209[ OH ] | F:ILE  21[ O  ] | 3.36 | D:ASN 379[ ND2] |
| F:ASP 414[ OD1] | 2.49 | A:LYS 412[ NZ ] |  |  |  | F:GLU 215[ OE1] | 2.97 | D:TYR 209[ OH ] |
| F:ASN 410[ OD1] | 2.94 | A:LYS 412[ NZ ] |  |  |  | F:ASP 419[ OD1] | 3.84 | D:ASN 389[ ND2] |
|  |  |  |  |  |  | F:ASN 424[ OD1] | 3.89 | D:ASN 424[ ND2] |
|  |  |  |  |  |  | F:GLU 426[ OE1] | 2.87 | D:LYS 387[ NZ ] |
| **Salt Bridges** | | | **Salt Bridges** | | | **Salt Bridges** | | |
| F:LYS 387[ NZ ] | 2.95 | A:GLU 426[ OE2] | D:ARG 405[ NE ] | 2.75 | A:GLU 102[ OE2] | F:ARG 405[ NE ] | 2.62 | D:GLU 102[ OE2] |
| F:GLU 102[ OE2] | 2.70 | A:ARG 405[ NE ] | D:ARG 405[ NH1] | 2.81 | A:GLU 403[ OE1] | F:ARG 405[ NH1] | 2.94 | D:GLU 403[ OE1] |
| F:GLU 403[ OE1] | 2.83 | A:ARG 405[ NH1] | D:ARG 405[ NH1] | 3.53 | A:GLU 403[ OE2] | F:ARG 405[ NH1] | 3.81 | D:GLU 403[ OE2] |
| F:GLU 403[ OE2] | 3.37 | A:ARG 405[ NH1] | D:ARG 405[ NH2] | 3.43 | A:GLU 403[ OE1] | F:ARG 405[ NH2] | 3.60 | D:GLU 102[ OE2] |
| F:GLU 102[ OE2] | 3.41 | A:ARG 405[ NH2] | D:ARG 405[ NH2] | 2.59 | A:GLU 403[ OE2] | F:ARG 405[ NH2] | 3.63 | D:GLU 403[ OE1] |
| F:GLU 403[ OE1] | 3.74 | A:ARG 405[ NH2] | D:ARG 405[ NH2] | 3.76 | A:GLU 102[ OE2] | F:ARG 405[ NH2] | 3.01 | D:GLU 403[ OE2] |
| F:GLU 403[ OE2] | 2.79 | A:ARG 405[ NH2] | D:LYS 412[ NZ ] | 2.52 | A:ASP 414[ OD1] | F:LYS 412[ NZ ] | 2.55 | D:ASP 414[ OD1] |
| F:ASP 414[ OD1] | 2.49 | A:LYS 412[ NZ ] | D:LYS 412[ NZ ] | 3.68 | A:ASP 414[ OD2] | F:LYS 412[ NZ ] | 3.51 | D:ASP 414[ OD2] |
| F:ASP 414[ OD2] | 3.44 | A:LYS 412[ NZ ] |  |  |  | F:ASP 419[ OD2] | 3.94 | D:LYS 307[ NZ ] |
|  |  |  |  |  |  | F:GLU 426[ OE1] | 2.87 | D:LYS 387[ NZ ] |
|  |  |  |  |  |  | F:ASP 438[ OD2] | 3.96 | D:ARG 435[ NH2] |

**Supplementary Table 5: Comparison between three interfaces in A/Almaty/32/1998 hemagglutinin**

| **PDB ID: 6MYA**  **Number of H-bonds: 18**  **Number of Salt bonds: 13**  **Interface area [Å2]: 1842.1** | | | **PDB ID: 6MYA**  **Number of H-bonds: 22**  **Number of Salt bonds: 13**  **Interface area [Å2]: 1926.5** | | | **PDB ID: 6MYA**  **Number of H-bonds: 24**  **Number of Salt bonds: 13**  **Interface area [Å2]: 1951.9** | | |
| --- | --- | --- | --- | --- | --- | --- | --- | --- |
| **Protomer 1** | [**Dist. [Å]**](javascript:openWindow('pi_ipage_atmdist.html',400,250);) | **Protomer 3** | **Protomer 2** | [**Dist. [Å]**](javascript:openWindow('pi_ipage_atmdist.html',400,250);) | **Protomer 3** | **Protomer 1** | [**Dist. [Å]**](javascript:openWindow('pi_ipage_atmdist.html',400,250);) | **Protomer 2** |
| C:SER 383[ OG ] | 3.70 | A:LEU  21[ O  ] | B:LYS  24[ NZ ] | 2.83 | A:SER 383[ OG ] | C:LYS  24[ NZ ] | 2.81 | B:SER 383[ OG ] |
| C:ASN 446[ ND2] | 3.02 | A:GLY 330[ O  ] | B:ALA 218[ N  ] | 2.89 | A:GLU 245[ OE1] | C:ALA 218[ N  ] | 3.06 | B:GLU 245[ OE1] |
| C:SER 442[ OG ] | 2.59 | A:LEU 331[ O  ] | B:ARG 228[ NH2] | 3.08 | A:SER 205[ O  ] | C:ARG 228[ NH2] | 3.15 | B:SER 205[ O  ] |
| C:LYS 387[ NZ ] | 2.70 | A:GLU 426[ OE2] | B:GLU 403[ N  ] | 3.46 | A:GLU 102[ OE1] | C:GLU 403[ N  ] | 3.42 | B:GLU 102[ OE2] |
| C:ARG 435[ NH2] | 3.18 | A:GLU 434[ OE1] | B:LYS 404[ N  ] | 2.86 | A:GLU 102[ OE1] | C:LYS 404[ N  ] | 2.84 | B:GLU 102[ OE2] |
| C:ARG 435[ NH2] | 3.45 | A:ASP 438[ OD2] | B:ARG 405[ N  ] | 3.02 | A:GLU 102[ OE2] | C:ARG 405[ N  ] | 3.04 | B:GLU 102[ OE1] |
| C:GLU 245[ OE1] | 3.41 | A:ALA 218[ N  ] | B:ARG 405[ NE ] | 2.72 | A:GLU 102[ OE2] | C:ARG 405[ NE ] | 2.72 | B:GLU 102[ OE1] |
| C:SER 205[ O  ] | 3.34 | A:ARG 228[ NH2] | B:ARG 405[ NH1] | 3.08 | A:GLU 398[ O  ] | C:ARG 405[ NH1] | 2.88 | B:GLU 398[ O  ] |
| C:GLU 102[ OE1] | 3.43 | A:GLU 403[ N  ] | B:ARG 405[ NH1] | 2.94 | A:GLU 403[ OE1] | C:ARG 405[ NH1] | 2.89 | B:GLU 403[ OE1] |
| C:GLU 102[ OE1] | 2.84 | A:LYS 404[ N  ] | B:ARG 405[ NH2] | 2.81 | A:GLU 403[ OE2] | C:ARG 405[ NH2] | 2.75 | B:GLU 403[ OE2] |
| C:GLU 102[ OE2] | 3.06 | A:ARG 405[ N  ] | B:ASN 408[ ND2] | 2.55 | A:GLU 105[ OE1] | C:ASN 408[ ND2] | 2.63 | B:GLU 105[ OE2] |
| C:GLU 102[ OE2] | 2.70 | A:ARG 405[ NE ] | B:LYS 412[ NZ ] | 2.80 | A:ASP 414[ OD1] | C:LYS 412[ NZ ] | 2.88 | B:ASP 414[ OD1] |
| C:GLU 403[ OE2] | 2.56 | A:ARG 405[ NH1] | B:LYS 412[ NZ ] | 2.88 | A:ASN 410[ OD1] | C:LYS 412[ NZ ] | 2.92 | B:ASN 410[ OD1] |
| C:GLU 398[ O  ] | 2.85 | A:ARG 405[ NH2] | B:TYR 423[ OH ] | 3.68 | A:ASN 424[ O  ] | C:TYR 423[ OH ] | 3.78 | B:ASN 424[ O  ] |
| C:GLU 403[ OE1] | 2.86 | A:ARG 405[ NH2] | B:LEU  21[ O  ] | 3.59 | A:SER 383[ OG ] | C:LEU  21[ O  ] | 2.57 | B:ASN 379[ ND2] |
| C:GLU 105[ OE2] | 2.73 | A:ASN 408[ ND2] | B:LEU  22[ O  ] | 3.45 | A:ASN 379[ ND2] | C:LEU  21[ O  ] | 3.88 | B:SER 383[ OG ] |
| C:ASN 410[ OD1] | 2.77 | A:LYS 412[ NZ ] | B:GLY 330[ O  ] | 3.13 | A:ASN 446[ ND2] | C:GLY 330[ O  ] | 2.98 | B:ASN 446[ ND2] |
| C:ASP 414[ OD1] | 2.81 | A:LYS 412[ NZ ] | B:GLY 330[ O  ] | 3.83 | A:SER 442[ OG ] | C:LEU 331[ O  ] | 3.86 | B:ASN 446[ ND2] |
|  |  |  | B:LEU 331[ O  ] | 2.49 | A:SER 442[ OG ] | C:LEU 331[ O  ] | 2.44 | B:SER 442[ OG ] |
|  |  |  | B:GLU 426[ OE2] | 2.83 | A:LYS 387[ NZ ] | C:ASN 424[ OD1] | 3.89 | B:ASN 424[ ND2] |
|  |  |  | B:GLU 434[ OE2] | 2.96 | A:ARG 435[ NH1] | C:GLU 426[ OE1] | 2.67 | B:LYS 387[ NZ ] |
|  |  |  | B:ASP 438[ OD2] | 3.03 | A:ARG 435[ NH1] | C:GLU 434[ OE2] | 2.92 | B:ARG 435[ NH1] |
|  |  |  |  |  |  | C:ASP 438[ OD2] | 3.03 | B:ARG 435[ NH1] |
|  |  |  |  |  |  | C:GLU 461[ O  ] | 3.41 | B:LYS 456[ NZ ] |
| **Salt Bridges** | | | **Salt Bridges** | | | **Salt Bridges** | | |
| C:LYS 387[ NZ ] | 2.70 | A:GLU 426[ OE2] | B:ARG 405[ NE ] | 2.72 | A:GLU 102[ OE2] | C:ARG 405[ NE ] | 2.72 | B:GLU 102[ OE1] |
| C:ARG 435[ NE ] | 3.82 | A:GLU 434[ OE1] | B:ARG 405[ NH1] | 3.51 | A:GLU 403[ OE2] | C:ARG 405[ NH1] | 2.89 | B:GLU 403[ OE1] |
| C:ARG 435[ NH2] | 3.18 | A:GLU 434[ OE1] | B:ARG 405[ NH1] | 2.94 | A:GLU 403[ OE1] | C:ARG 405[ NH1] | 3.54 | B:GLU 403[ OE2] |
| C:ARG 435[ NE ] | 3.89 | A:GLU 434[ OE2] | B:ARG 405[ NH2] | 2.81 | A:GLU 403[ OE2] | C:ARG 405[ NH2] | 3.52 | B:GLU 102[ OE1] |
| C:ARG 435[ NH2] | 3.45 | A:ASP 438[ OD2] | B:ARG 405[ NH2] | 3.66 | A:GLU 102[ OE2] | C:ARG 405[ NH2] | 3.63 | B:GLU 403[ OE1] |
| C:GLU 102[ OE2] | 2.70 | A:ARG 405[ NE ] | B:ARG 405[ NH2] | 3.71 | A:GLU 403[ OE1] | C:ARG 405[ NH2] | 2.75 | B:GLU 403[ OE2] |
| C:GLU 403[ OE1] | 3.50 | A:ARG 405[ NH1] | B:LYS 412[ NZ ] | 2.80 | A:ASP 414[ OD1] | C:LYS 412[ NZ ] | 2.88 | B:ASP 414[ OD1] |
| C:GLU 403[ OE2] | 2.56 | A:ARG 405[ NH1] | B:LYS 412[ NZ ] | 3.47 | A:ASP 414[ OD2] | C:LYS 412[ NZ ] | 3.63 | B:ASP 414[ OD2] |
| C:GLU 102[ OE2] | 3.70 | A:ARG 405[ NH1] | B:GLU 426[ OE2] | 2.83 | A:LYS 387[ NZ ] | C:GLU 426[ OE1] | 2.67 | B:LYS 387[ NZ ] |
| C:GLU 403[ OE1] | 2.86 | A:ARG 405[ NH2] | B:GLU 434[ OE1] | 3.77 | A:ARG 435[ NE ] | C:GLU 434[ OE1] | 3.85 | B:ARG 435[ NE ] |
| C:GLU 403[ OE2] | 3.42 | A:ARG 405[ NH2] | B:GLU 434[ OE2] | 3.42 | A:ARG 435[ NE ] | C:GLU 434[ OE2] | 3.61 | B:ARG 435[ NE ] |
| C:ASP 414[ OD1] | 2.81 | A:LYS 412[ NZ ] | B:GLU 434[ OE2] | 2.96 | A:ARG 435[ NH1] | C:GLU 434[ OE2] | 2.92 | B:ARG 435[ NH1] |
| C:ASP 414[ OD2] | 3.51 | A:LYS 412[ NZ ] | B:ASP 438[ OD2] | 3.03 | A:ARG 435[ NH1] | C:ASP 438[ OD2] | 3.03 | B:ARG 435[ NH1] |

**Supplementary Table 6: Comparison between three interfaces in A/Netherlands/002P1/1951 hemagglutinin**

| **PDB ID: 6N41**  **Number of H-bonds: 23**  **Number of Salt bonds: 12**  **Interface area [Å2]: 1988.2** | | | **PDB ID: 6N41**  **Number of H-bonds: 22**  **Number of Salt bonds: 12**  **Interface area [Å2]: 2105.3** | | | **PDB ID: 6N41**  **Number of H-bonds: 26**  **Number of Salt bonds: 12**  **Interface area [Å2]: 2187.1** | | |
| --- | --- | --- | --- | --- | --- | --- | --- | --- |
| **Protomer 1** | [**Dist. [Å]**](javascript:openWindow('pi_ipage_atmdist.html',400,250);) | **Protomer 2** | **Protomer 3** | [**Dist. [Å]**](javascript:openWindow('pi_ipage_atmdist.html',400,250);) | **Protomer 2** | **Protomer 1** | [**Dist. [Å]**](javascript:openWindow('pi_ipage_atmdist.html',400,250);) | **Protomer 3** |
| C:ASN 379[ ND2] | 3.58 | A:THR  20[ O  ] | B:GLU 218[ N  ] | 3.60 | A:GLU 245[ OE1] | C:GLU 218[ N  ] | 3.52 | B:GLU 245[ OE2] |
| C:ASN 379[ ND2] | 2.86 | A:VAL  21[ O  ] | B:ARG 228[ NH2] | 3.20 | A:SER 205[ O  ] | C:ARG 228[ NH2] | 3.35 | B:SER 205[ O  ] |
| C:ARG 211[ N  ] | 3.83 | A:GLU 215[ OE1] | B:LYS 401[ NZ ] | 3.52 | A:ASN 207[ O  ] | C:LYS 310[ NZ ] | 3.17 | B:GLN 391[ OE1] |
| C:ASN 446[ ND2] | 3.11 | A:GLY 330[ O  ] | B:LYS 404[ N  ] | 2.34 | A:GLU 102[ OE2] | C:LYS 404[ N  ] | 2.40 | B:GLU 102[ OE2] |
| C:SER 442[ OG ] | 2.70 | A:LEU 331[ O  ] | B:ARG 405[ N  ] | 3.30 | A:GLU 102[ OE2] | C:ARG 405[ N  ] | 3.39 | B:GLU 102[ OE2] |
| C:ASN 446[ ND2] | 3.75 | A:LEU 331[ O  ] | B:ARG 405[ NE ] | 2.49 | A:GLU 102[ OE1] | C:ARG 405[ NE ] | 2.54 | B:GLU 102[ OE1] |
| C:GLN 106[ NE2] | 3.15 | A:LYS 401[ O  ] | B:ARG 405[ NH1] | 2.73 | A:GLU 403[ OE1] | C:ARG 405[ NH1] | 2.82 | B:GLU 398[ O  ] |
| C:ASN 389[ ND2] | 3.59 | A:ASP 419[ OD1] | B:ARG 405[ NH1] | 2.90 | A:GLU 398[ O  ] | C:ARG 405[ NH1] | 2.78 | B:GLU 403[ OE1] |
| C:LYS 307[ NZ ] | 3.07 | A:ASP 419[ OD2] | B:ARG 405[ NH2] | 2.80 | A:GLU 403[ OE2] | C:ARG 405[ NH2] | 2.81 | B:GLU 403[ OE2] |
| C:ARG 435[ NH2] | 3.26 | A:ASP 438[ OD2] | B:ASN 408[ ND2] | 2.84 | A:GLU 105[ OE2] | C:ASN 408[ ND2] | 2.75 | B:GLU 105[ OE2] |
| C:ASN 209[ OD1] | 3.21 | A:ARG 219[ NH1] | B:LYS 412[ NZ ] | 2.74 | A:ASP 414[ OD1] | C:LYS 412[ NZ ] | 2.72 | B:VAL 395[ O  ] |
| C:SER 205[ O  ] | 3.20 | A:ARG 228[ NH2] | B:LYS 412[ NZ ] | 2.87 | A:VAL 395[ O  ] | C:LYS 412[ NZ ] | 2.83 | B:ASP 414[ OD1] |
| C:GLU 102[ OE2] | 2.47 | A:LYS 404[ N  ] | B:LYS 412[ NZ ] | 2.77 | A:ASN 410[ OD1] | C:LYS 412[ NZ ] | 2.76 | B:ASN 410[ OD1] |
| C:GLU 102[ OE2] | 2.99 | A:ARG 405[ N  ] | B:TYR 423[ OH ] | 2.57 | A:VAL 384[ O  ] | C:TYR 423[ OH ] | 2.63 | B:VAL 384[ O  ] |
| C:GLU 102[ OE1] | 2.51 | A:ARG 405[ NE ] | B:THR  20[ O  ] | 3.33 | A:ASN 379[ ND2] | C:THR  20[ O  ] | 3.54 | B:ASN 379[ ND2] |
| C:GLU 403[ OE1] | 2.84 | A:ARG 405[ NH1] | B:VAL  21[ O  ] | 2.70 | A:ASN 379[ ND2] | C:VAL  21[ O  ] | 2.85 | B:ASN 379[ ND2] |
| C:GLU 398[ O  ] | 2.97 | A:ARG 405[ NH1] | B:ASP  96[ OD2] | 2.85 | A:ASN 209[ ND2] | C:GLY 330[ O  ] | 3.27 | B:ASN 446[ ND2] |
| C:GLU 403[ OE2] | 2.71 | A:ARG 405[ NH2] | B:GLY 330[ O  ] | 3.16 | A:ASN 446[ ND2] | C:LEU 331[ O  ] | 2.72 | B:SER 442[ OG ] |
| C:GLU 105[ OE2] | 2.53 | A:ASN 408[ ND2] | B:LEU 331[ O  ] | 3.82 | A:ASN 446[ ND2] | C:LEU 331[ O  ] | 3.78 | B:ASN 446[ ND2] |
| C:ASP 414[ OD1] | 2.78 | A:LYS 412[ NZ ] | B:LEU 331[ O  ] | 2.66 | A:SER 442[ OG ] | C:LYS 401[ O  ] | 3.09 | B:GLN 106[ NE2] |
| C:ASN 410[ OD1] | 2.59 | A:LYS 412[ NZ ] | B:LYS 401[ O  ] | 3.65 | A:GLN 106[ NE2] | C:ASP 415[ OD1] | 3.15 | B:GLN 391[ NE2] |
| C:VAL 395[ O  ] | 2.73 | A:LYS 412[ NZ ] | B:ASP 438[ OD2] | 2.99 | A:ARG 435[ NH2] | C:ASP 419[ OD1] | 2.78 | B:ASN 389[ ND2] |
| C:VAL 384[ O  ] | 2.56 | A:TYR 423[ OH ] |  |  |  | C:ASP 419[ OD2] | 3.38 | B:GLN 391[ N  ] |
|  |  |  |  |  |  | C:ASP 419[ OD2] | 2.97 | B:LYS 307[ NZ ] |
|  |  |  |  |  |  | C:ASN 424[ OD1] | 3.90 | B:ASN 424[ ND2] |
|  |  |  |  |  |  | C:ASP 438[ OD2] | 2.87 | B:ARG 435[ NH2] |
| **Salt Bridges** | | | **Salt Bridges** | | | **Salt Bridges** | | |
| C:LYS 307[ NZ ] | 3.07 | A:ASP 419[ OD2] | B:ARG 405[ NE ] | 2.49 | A:GLU 102[ OE1] | C:ARG 405[ NE ] | 2.54 | B:GLU 102[ OE1] |
| C:LYS 387[ NZ ] | 3.73 | A:GLU 426[ OE2] | B:ARG 405[ NH1] | 2.73 | A:GLU 403[ OE1] | C:ARG 405[ NH1] | 2.78 | B:GLU 403[ OE1] |
| C:ARG 435[ NH1] | 3.94 | A:ASP 438[ OD2] | B:ARG 405[ NH1] | 3.53 | A:GLU 403[ OE2] | C:ARG 405[ NH1] | 3.57 | B:GLU 403[ OE2] |
| C:ARG 435[ NH2] | 3.26 | A:ASP 438[ OD2] | B:ARG 405[ NH2] | 3.13 | A:GLU 102[ OE1] | C:ARG 405[ NH2] | 3.24 | B:GLU 102[ OE1] |
| C:GLU 102[ OE1] | 2.51 | A:ARG 405[ NE ] | B:ARG 405[ NH2] | 3.56 | A:GLU 403[ OE1] | C:ARG 405[ NH2] | 3.58 | B:GLU 403[ OE1] |
| C:GLU 403[ OE1] | 2.84 | A:ARG 405[ NH1] | B:ARG 405[ NH2] | 2.80 | A:GLU 403[ OE2] | C:ARG 405[ NH2] | 2.81 | B:GLU 403[ OE2] |
| C:GLU 403[ OE2] | 3.40 | A:ARG 405[ NH1] | B:LYS 412[ NZ ] | 2.74 | A:ASP 414[ OD1] | C:LYS 412[ NZ ] | 2.83 | B:ASP 414[ OD1] |
| C:GLU 403[ OE1] | 3.69 | A:ARG 405[ NH2] | B:LYS 412[ NZ ] | 3.37 | A:ASP 414[ OD2] | C:LYS 412[ NZ ] | 3.49 | B:ASP 414[ OD2] |
| C:GLU 403[ OE2] | 2.71 | A:ARG 405[ NH2] | B:ASP 419[ OD2] | 3.32 | A:LYS 307[ NZ ] | C:ASP 419[ OD2] | 2.97 | B:LYS 307[ NZ ] |
| C:GLU 102[ OE1] | 3.23 | A:ARG 405[ NH2] | B:GLU 426[ OE2] | 3.61 | A:LYS 387[ NZ ] | C:GLU 426[ OE1] | 3.66 | B:LYS 387[ NZ ] |
| C:ASP 414[ OD2] | 3.56 | A:LYS 412[ NZ ] | B:ASP 438[ OD2] | 3.48 | A:ARG 435[ NH1] | C:ASP 438[ OD2] | 2.87 | B:ARG 435[ NH2] |
| C:ASP 414[ OD1] | 2.78 | A:LYS 412[ NZ ] | B:ASP 438[ OD2] | 2.99 | A:ARG 435[ NH2] | C:ASP 438[ OD2] | 3.44 | B:ARG 435[ NH1] |

**Supplementary Table 7: Comparison between three interfaces in A/Hickox/JY2/1940 hemagglutinin**

| **PDB ID: 6ONA**  **Number of H-bonds: 26**  **Number of Salt bonds: 11**  **Interface area [Å2]: 2071.3** | | | **PDB ID: 6ONA**  **Number of H-bonds: 25**  **Number of Salt bonds: 11**  **Interface area [Å2]: 2172.6** | | | **PDB ID: 6ONA**  **Number of H-bonds: 25**  **Number of Salt bonds: 11**  **Interface area [Å2]: 2162.2** | | |
| --- | --- | --- | --- | --- | --- | --- | --- | --- |
| **Protomer 1** | [**Dist. [Å]**](javascript:openWindow('pi_ipage_atmdist.html',400,250);) | **Protomer 2** | **Protomer 3** | [**Dist. [Å]**](javascript:openWindow('pi_ipage_atmdist.html',400,250);) | **Protomer 2** | **Protomer 1** | [**Dist. [Å]**](javascript:openWindow('pi_ipage_atmdist.html',400,250);) | **Protomer 3** |
| C:ASN 379[ ND2] | 3.56 | A:THR  20[ O  ] | B:ARG 228[ NH2] | 3.48 | A:SER 205[ O  ] | C:ARG 219[ NH2] | 2.69 | B:ASN 209[ OD1] |
| C:ASN 379[ ND2] | 2.92 | A:VAL  21[ O  ] | B:GLU 403[ N  ] | 3.52 | A:GLU 102[ OE1] | C:ARG 228[ NE ] | 3.88 | B:SER 206[ O  ] |
| C:ARG 211[ NH1] | 3.46 | A:ILE 216[ O  ] | B:LYS 404[ N  ] | 2.82 | A:GLU 102[ OE1] | C:ARG 228[ NH2] | 3.67 | B:SER 206[ O  ] |
| C:ASN 446[ ND2] | 3.70 | A:GLY 330[ O  ] | B:ARG 405[ N  ] | 3.22 | A:GLU 102[ OE2] | C:ARG 228[ NH2] | 3.46 | B:SER 205[ O  ] |
| C:SER 442[ OG ] | 2.72 | A:LEU 331[ O  ] | B:ARG 405[ NE ] | 2.79 | A:GLU 102[ OE2] | C:GLU 403[ N  ] | 3.49 | B:GLU 102[ OE1] |
| C:ASN 446[ ND2] | 3.56 | A:LEU 331[ O  ] | B:ARG 405[ NH1] | 3.00 | A:GLU 398[ O  ] | C:LYS 404[ N  ] | 2.85 | B:GLU 102[ OE1] |
| C:GLN 106[ NE2] | 3.19 | A:LYS 401[ O  ] | B:ARG 405[ NH1] | 2.87 | A:GLU 403[ OE1] | C:ARG 405[ N  ] | 3.09 | B:GLU 102[ OE2] |
| C:ASN 389[ ND2] | 3.01 | A:ASP 419[ OD1] | B:ARG 405[ NH2] | 2.70 | A:GLU 403[ OE2] | C:ARG 405[ NE ] | 2.70 | B:GLU 102[ OE2] |
| C:GLN 391[ NE2] | 2.50 | A:ASP 419[ OD2] | B:ASN 408[ ND2] | 2.61 | A:GLU 105[ OE2] | C:ARG 405[ NH1] | 2.92 | B:GLU 403[ OE2] |
| C:ASN 424[ ND2] | 3.90 | A:ASN 424[ OD1] | B:LYS 412[ NZ ] | 2.87 | A:VAL 395[ O  ] | C:ARG 405[ NH2] | 2.91 | B:GLU 398[ O  ] |
| C:LYS 387[ NZ ] | 3.24 | A:GLU 426[ OE2] | B:LYS 412[ NZ ] | 2.85 | A:ASP 414[ OD1] | C:ARG 405[ NH2] | 2.99 | B:GLU 403[ OE1] |
| C:ARG 435[ NH2] | 2.85 | A:ASP 438[ OD2] | B:LYS 412[ NZ ] | 2.75 | A:ASN 410[ OD1] | C:ASN 408[ ND2] | 2.89 | B:GLU 105[ OE1] |
| C:GLU 245[ OE2] | 3.28 | A:GLU 218[ N  ] | B:TYR 423[ OH ] | 2.61 | A:VAL 384[ O  ] | C:LYS 412[ NZ ] | 2.82 | B:ASP 414[ OD1] |
| C:SER 205[ O  ] | 3.09 | A:ARG 228[ NH2] | B:THR  20[ O  ] | 3.65 | A:ASN 379[ ND2] | C:LYS 412[ NZ ] | 2.90 | B:VAL 395[ O  ] |
| C:GLU 102[ OE1] | 3.42 | A:GLU 403[ N  ] | B:VAL  21[ O  ] | 2.89 | A:ASN 379[ ND2] | C:LYS 412[ NZ ] | 2.72 | B:ASN 410[ OD1] |
| C:GLU 102[ OE1] | 2.75 | A:LYS 404[ N  ] | B:GLY 330[ O  ] | 3.67 | A:ASN 446[ ND2] | C:TYR 423[ OH ] | 2.63 | B:VAL 384[ O  ] |
| C:GLU 102[ OE2] | 3.11 | A:ARG 405[ N  ] | B:LEU 331[ O  ] | 2.76 | A:SER 442[ OG ] | C:THR  20[ O  ] | 3.50 | B:ASN 379[ ND2] |
| C:GLU 102[ OE2] | 2.80 | A:ARG 405[ NE ] | B:LEU 331[ O  ] | 3.50 | A:ASN 446[ ND2] | C:VAL  21[ O  ] | 2.79 | B:ASN 379[ ND2] |
| C:GLU 403[ OE1] | 2.78 | A:ARG 405[ NH1] | B:LYS 401[ O  ] | 3.08 | A:GLN 106[ NE2] | C:GLY 330[ O  ] | 3.52 | B:ASN 446[ ND2] |
| C:GLU 398[ O  ] | 2.92 | A:ARG 405[ NH2] | B:ASP 415[ O  ] | 3.62 | A:GLN 391[ NE2] | C:LEU 331[ O  ] | 2.60 | B:SER 442[ OG ] |
| C:GLU 403[ OE2] | 2.92 | A:ARG 405[ NH2] | B:ASP 419[ OD1] | 2.79 | A:ASN 389[ ND2] | C:LEU 331[ O  ] | 3.60 | B:ASN 446[ ND2] |
| C:GLU 105[ OE2] | 2.63 | A:ASN 408[ ND2] | B:ASP 419[ OD2] | 2.47 | A:GLN 391[ NE2] | C:LYS 401[ O  ] | 3.02 | B:GLN 106[ NE2] |
| C:ASP 414[ OD1] | 2.84 | A:LYS 412[ NZ ] | B:ASN 424[ OD1] | 3.77 | A:ASN 424[ ND2] | C:ASP 419[ OD2] | 2.59 | B:GLN 391[ NE2] |
| C:VAL 395[ O  ] | 2.88 | A:LYS 412[ NZ ] | B:GLU 426[ OE1] | 2.80 | A:LYS 387[ NZ ] | C:GLU 426[ OE1] | 2.76 | B:LYS 387[ NZ ] |
| C:ASN 410[ OD1] | 2.85 | A:LYS 412[ NZ ] | B:ASP 438[ OD2] | 3.06 | A:ARG 435[ NH2] | C:ASP 438[ OD2] | 2.58 | B:ARG 435[ NH1] |
| C:VAL 384[ O  ] | 2.67 | A:TYR 423[ OH ] |  |  |  |  |  |  |
| **Salt Bridges** | | | **Salt Bridges** | | | **Salt Bridges** | | |
| C:LYS 307[ NZ ] | 3.17 | A:ASP 419[ OD2] | B:ARG 405[ NE ] | 2.79 | A:GLU 102[ OE2] | C:ARG 405[ NE ] | 2.70 | B:GLU 102[ OE2] |
| C:LYS 387[ NZ ] | 3.24 | A:GLU 426[ OE2] | B:ARG 405[ NH1] | 2.87 | A:GLU 403[ OE1] | C:ARG 405[ NH1] | 3.51 | B:GLU 102[ OE2] |
| C:ARG 435[ NH2] | 2.85 | A:ASP 438[ OD2] | B:ARG 405[ NH1] | 3.42 | A:GLU 403[ OE2] | C:ARG 405[ NH1] | 2.92 | B:GLU 403[ OE2] |
| C:GLU 102[ OE2] | 2.80 | A:ARG 405[ NE ] | B:ARG 405[ NH2] | 3.68 | A:GLU 403[ OE1] | C:ARG 405[ NH1] | 3.76 | B:GLU 403[ OE1] |
| C:GLU 102[ OE2] | 3.73 | A:ARG 405[ NH1] | B:ARG 405[ NH2] | 3.61 | A:GLU 102[ OE2] | C:ARG 405[ NH2] | 3.63 | B:GLU 403[ OE2] |
| C:GLU 403[ OE1] | 2.78 | A:ARG 405[ NH1] | B:ARG 405[ NH2] | 2.70 | A:GLU 403[ OE2] | C:ARG 405[ NH2] | 2.99 | B:GLU 403[ OE1] |
| C:GLU 403[ OE2] | 3.50 | A:ARG 405[ NH1] | B:LYS 412[ NZ ] | 2.85 | A:ASP 414[ OD1] | C:LYS 412[ NZ ] | 2.82 | B:ASP 414[ OD1] |
| C:GLU 403[ OE1] | 3.71 | A:ARG 405[ NH2] | B:LYS 412[ NZ ] | 3.56 | A:ASP 414[ OD2] | C:LYS 412[ NZ ] | 3.54 | B:ASP 414[ OD2] |
| C:GLU 403[ OE2] | 2.92 | A:ARG 405[ NH2] | B:ASP 419[ OD2] | 3.13 | A:LYS 307[ NZ ] | C:ASP 419[ OD2] | 2.96 | B:LYS 307[ NZ ] |
| C:ASP 414[ OD1] | 2.84 | A:LYS 412[ NZ ] | B:GLU 426[ OE1] | 2.80 | A:LYS 387[ NZ ] | C:GLU 426[ OE1] | 2.76 | B:LYS 387[ NZ ] |
| C:ASP 414[ OD2] | 3.50 | A:LYS 412[ NZ ] | B:ASP 438[ OD2] | 3.06 | A:ARG 435[ NH2] | C:ASP 438[ OD2] | 2.58 | B:ARG 435[ NH1] |

**Supplementary Table 8: Comparison between three interfaces in A/Melbourne/1/1946 hemagglutinin**

| **PDB ID: 6OSR**  **Number of H-bonds: 19**  **Number of Salt bonds: 11**  **Interface area [Å2]: 1858.0** | | | **PDB ID: 6OSR**  **Number of H-bonds: 19**  **Number of Salt bonds: 10**  **Interface area [Å2]: 1898.3** | | | **PDB ID: 6OSR**  **Number of H-bonds: 21**  **Number of Salt bonds: 10**  **Interface area [Å2]: 1909.1** | | |
| --- | --- | --- | --- | --- | --- | --- | --- | --- |
| **Protomer 2** | [**Dist. [Å]**](javascript:openWindow('pi_ipage_atmdist.html',400,250);) | **Protomer 3** | **Protomer 1** | [**Dist. [Å]**](javascript:openWindow('pi_ipage_atmdist.html',400,250);) | **Protomer 3** | **Protomer 1** | [**Dist. [Å]**](javascript:openWindow('pi_ipage_atmdist.html',400,250);) | **Protomer 2** |
| B:ARG 219[ NE ] | 3.25 | A:ASN 209[ OD1] | C:ASN 446[ ND2] | 3.47 | A:GLY 330[ O  ] | C:GLU  23[ N  ] | 3.88 | B:ASN 379[ OD1] |
| B:ARG 228[ NH2] | 3.66 | A:SER 205[ O  ] | C:SER 442[ OG ] | 2.57 | A:LEU 331[ O  ] | C:GLU 218[ N  ] | 3.89 | B:GLU 245[ OE1] |
| B:LYS 404[ N  ] | 2.62 | A:GLU 102[ OE1] | C:ASN 446[ ND2] | 3.78 | A:LEU 331[ O  ] | C:ARG 219[ NE ] | 3.76 | B:ASN 209[ OD1] |
| B:ARG 405[ N  ] | 3.19 | A:GLU 102[ OE2] | C:LYS 397[ NZ ] | 2.86 | A:ARG 405[ O  ] | C:ARG 228[ NH2] | 3.61 | B:SER 205[ O  ] |
| B:ARG 405[ NE ] | 2.52 | A:GLU 102[ OE2] | C:ASN 424[ ND2] | 3.86 | A:ASN 424[ OD1] | C:GLU 403[ N  ] | 3.63 | B:GLU 102[ OE1] |
| B:ARG 405[ NH1] | 2.83 | A:GLU 403[ OE2] | C:LYS 387[ NZ ] | 2.37 | A:GLU 426[ OE1] | C:LYS 404[ N  ] | 2.85 | B:GLU 102[ OE1] |
| B:ARG 405[ NH2] | 2.85 | A:GLU 403[ OE1] | C:ARG 435[ NH2] | 3.12 | A:ASP 438[ OD2] | C:ARG 405[ N  ] | 3.62 | B:GLU 102[ OE1] |
| B:ARG 405[ NH2] | 3.18 | A:GLU 398[ O  ] | C:ASN 209[ OD1] | 3.70 | A:ARG 219[ NE ] | C:ARG 405[ N  ] | 3.27 | B:GLU 102[ OE2] |
| B:ASN 408[ ND2] | 2.60 | A:GLU 105[ OE2] | C:SER 205[ O  ] | 3.38 | A:ARG 228[ NH2] | C:ARG 405[ NE ] | 2.76 | B:GLU 102[ OE2] |
| B:LYS 412[ NZ ] | 2.92 | A:ASN 410[ OD1] | C:GLU 102[ OE1] | 3.47 | A:GLU 403[ N  ] | C:ARG 405[ NH1] | 2.72 | B:GLU 403[ OE1] |
| B:LYS 412[ NZ ] | 2.65 | A:ASP 414[ OD1] | C:GLU 102[ OE1] | 2.86 | A:LYS 404[ N  ] | C:ARG 405[ NH2] | 3.04 | B:GLU 398[ O  ] |
| B:THR  20[ O  ] | 3.44 | A:ASN 379[ ND2] | C:GLU 102[ OE2] | 3.33 | A:ARG 405[ N  ] | C:ARG 405[ NH2] | 2.82 | B:GLU 403[ OE2] |
| B:VAL  21[ O  ] | 3.49 | A:ASN 379[ ND2] | C:GLU 102[ OE2] | 2.90 | A:ARG 405[ NE ] | C:ASN 408[ ND2] | 2.76 | B:GLU 105[ OE2] |
| B:GLU 215[ OE1] | 3.88 | A:ARG 211[ N  ] | C:GLU 403[ OE1] | 2.57 | A:ARG 405[ NH1] | C:LYS 412[ NZ ] | 2.75 | B:ASN 410[ OD1] |
| B:GLY 330[ O  ] | 3.07 | A:ASN 446[ ND2] | C:GLU 403[ OE2] | 2.68 | A:ARG 405[ NH2] | C:LYS 412[ NZ ] | 2.70 | B:ASP 414[ OD1] |
| B:LEU 331[ O  ] | 2.57 | A:SER 442[ OG ] | C:GLU 398[ O  ] | 3.37 | A:ARG 405[ NH2] | C:ILE 216[ O  ] | 3.74 | B:ARG 211[ NH1] |
| B:LEU 331[ O  ] | 3.53 | A:ASN 446[ ND2] | C:GLU 105[ OE2] | 2.91 | A:ASN 408[ ND2] | C:GLY 330[ O  ] | 3.02 | B:ASN 446[ ND2] |
| B:GLU 426[ OE2] | 2.60 | A:LYS 387[ NZ ] | C:ASP 414[ OD1] | 2.72 | A:LYS 412[ NZ ] | C:LEU 331[ O  ] | 3.84 | B:ASN 446[ ND2] |
| B:ASP 438[ OD2] | 3.31 | A:ARG 435[ NH2] | C:ASN 410[ OD1] | 2.98 | A:LYS 412[ NZ ] | C:LEU 331[ O  ] | 2.40 | B:SER 442[ OG ] |
|  |  |  |  |  |  | C:GLU 426[ OE1] | 2.92 | B:LYS 387[ NZ ] |
|  |  |  |  |  |  | C:ASP 438[ OD2] | 3.15 | B:ARG 435[ NH2] |
| **Salt Bridges** | | | **Salt Bridges** | | | **Salt Bridges** | | |
| B:ARG 405[ NE ] | 2.52 | A:GLU 102[ OE2] | C:LYS 387[ NZ ] | 2.37 | A:GLU 426[ OE1] | C:ARG 405[ NE ] | 2.76 | B:GLU 102[ OE2] |
| B:ARG 405[ NH1] | 3.35 | A:GLU 102[ OE2] | C:ARG 435[ NH2] | 3.12 | A:ASP 438[ OD2] | C:ARG 405[ NH1] | 2.72 | B:GLU 403[ OE1] |
| B:ARG 405[ NH1] | 3.53 | A:GLU 403[ OE1] | C:GLU 102[ OE2] | 2.90 | A:ARG 405[ NE ] | C:ARG 405[ NH1] | 3.57 | B:GLU 403[ OE2] |
| B:ARG 405[ NH1] | 2.83 | A:GLU 403[ OE2] | C:GLU 403[ OE1] | 2.57 | A:ARG 405[ NH1] | C:ARG 405[ NH1] | 3.30 | B:GLU 102[ OE2] |
| B:ARG 405[ NH2] | 2.85 | A:GLU 403[ OE1] | C:GLU 403[ OE2] | 3.68 | A:ARG 405[ NH1] | C:ARG 405[ NH2] | 3.44 | B:GLU 403[ OE1] |
| B:ARG 405[ NH2] | 3.69 | A:GLU 403[ OE2] | C:GLU 102[ OE2] | 3.52 | A:ARG 405[ NH1] | C:ARG 405[ NH2] | 2.82 | B:GLU 403[ OE2] |
| B:LYS 412[ NZ ] | 2.65 | A:ASP 414[ OD1] | C:GLU 403[ OE1] | 3.19 | A:ARG 405[ NH2] | C:LYS 412[ NZ ] | 2.70 | B:ASP 414[ OD1] |
| B:LYS 412[ NZ ] | 3.20 | A:ASP 414[ OD2] | C:GLU 403[ OE2] | 2.68 | A:ARG 405[ NH2] | C:LYS 412[ NZ ] | 3.53 | B:ASP 414[ OD2] |
| B:GLU 426[ OE2] | 2.60 | A:LYS 387[ NZ ] | C:ASP 414[ OD1] | 2.72 | A:LYS 412[ NZ ] | C:GLU 426[ OE1] | 2.92 | B:LYS 387[ NZ ] |
| B:ASP 438[ OD2] | 3.97 | A:ARG 435[ NH1] | C:ASP 414[ OD2] | 3.23 | A:LYS 412[ NZ ] | C:ASP 438[ OD2] | 3.15 | B:ARG 435[ NH2] |
| B:ASP 438[ OD2] | 3.31 | A:ARG 435[ NH2] |  |  |  |  |  |  |

**Supplementary Table 9: Comparison between three interfaces in A/Fort Monmouth/1/1947 hemagglutinin**

| **PDB ID: 7JPD**  **Number of H-bonds: 16**  **Number of Salt bonds: 8**  **Interface area [Å2]: 1658.9** | | | **PDB ID: 7JPD**  **Number of H-bonds: 14**  **Number of Salt bonds: 10**  **Interface area [Å2]: 1681.9** | | | **PDB ID: 7JPD**  **Number of H-bonds: 13**  **Number of Salt bonds: 9**  **Interface area [Å2]: 1606.4** | | |
| --- | --- | --- | --- | --- | --- | --- | --- | --- |
| **Protomer 1** | [**Dist. [Å]**](javascript:openWindow('pi_ipage_atmdist.html',400,250);) | **Protomer 2** | **Protomer 3** | [**Dist. [Å]**](javascript:openWindow('pi_ipage_atmdist.html',400,250);) | **Protomer 1** | **Protomer 3** | [**Dist. [Å]**](javascript:openWindow('pi_ipage_atmdist.html',400,250);) | **Protomer 2** |
| d:ARG 405[ NH1] | 2.92 | a:GLU 398[ O  ] | e:ARG 405[ NH1] | 2.78 | d:GLU 398[ O  ] | e:ASN 446[ ND2] | 3.31 | a:GLY 330[ O  ] |
| d:ARG 405[ NH1] | 2.92 | a:GLU 403[ OE1] | e:ARG 405[ NH1] | 2.91 | d:GLU 403[ OE1] | e:SER 442[ OG ] | 2.46 | a:LEU 331[ O  ] |
| d:ARG 405[ NH2] | 2.90 | a:GLU 403[ OE2] | e:ARG 405[ NH2] | 2.65 | d:GLU 403[ OE2] | e:LYS 387[ NZ ] | 2.94 | a:GLU 426[ OE2] |
| d:LYS 412[ NZ ] | 2.67 | a:ASP 414[ OD1] | e:LYS 412[ NZ ] | 3.23 | d:ASN 410[ OD1] | e:ARG 435[ NH1] | 2.81 | a:ASP 438[ OD2] |
| d:LYS 412[ NZ ] | 2.88 | a:ASN 410[ OD1] | e:LYS 412[ NZ ] | 2.86 | d:VAL 395[ O  ] | e:GLU 398[ O  ] | 2.88 | a:ARG 405[ NH1] |
| d:LYS 412[ NZ ] | 3.30 | a:VAL 395[ O  ] | e:TYR 423[ OH ] | 2.78 | d:VAL 384[ O  ] | e:GLU 403[ OE1] | 2.89 | a:ARG 405[ NH1] |
| d:TYR 423[ OH ] | 2.82 | a:VAL 384[ O  ] | e:GLY 330[ O  ] | 3.16 | d:ASN 446[ ND2] | e:GLU 403[ OE2] | 2.84 | a:ARG 405[ NH2] |
| d:GLY 330[ O  ] | 3.16 | a:ASN 446[ ND2] | e:LEU 331[ O  ] | 2.53 | d:SER 442[ OG ] | e:VAL 395[ O  ] | 2.79 | a:LYS 412[ NZ ] |
| d:LEU 331[ O  ] | 2.49 | a:SER 442[ OG ] | e:ASP 438[ OD2] | 2.84 | d:ARG 435[ NH2] | e:ASN 410[ OD1] | 3.33 | a:LYS 412[ NZ ] |
| d:LEU 331[ O  ] | 3.69 | a:ASN 446[ ND2] | e:GLU 461[ O  ] | 2.46 | d:ARG 456[ NH1] | e:VAL 384[ O  ] | 3.04 | a:TYR 423[ OH ] |
| d:GLU 426[ OE2] | 3.56 | a:LYS 387[ NZ ] | E:ARG 219[ NE ] | 3.59 | D:ASN 209[ OD1] | E:ASN 209[ ND2] | 3.69 | A:GLU 215[ OE1] |
| d:ASP 438[ OD2] | 2.76 | a:ARG 435[ NH1] | E:ARG 228[ NE ] | 3.88 | D:SER 206[ O  ] | E:ASN 209[ OD1] | 3.23 | A:ARG 219[ NE ] |
| d:GLY 463[ O  ] | 3.87 | a:ASN 453[ ND2] | E:ARG 228[ NH2] | 3.25 | D:SER 205[ O  ] | E:SER 205[ O  ] | 3.35 | A:ARG 228[ NH2] |
| D:ARG 219[ NE ] | 3.36 | A:ASN 209[ OD1] | E:GLU 215[ OE1] | 3.85 | D:ARG 211[ N  ] |  |  |  |
| D:ARG 228[ NH2] | 3.20 | A:SER 205[ O  ] |  |  |  |  |  |  |
| D:GLU 215[ OE2] | 3.89 | A:ARG 211[ N  ] |  |  |  |  |  |  |
| **Salt Bridges** | | | **Salt Bridges** | | | **Salt Bridges** | | |
| d:ARG 405[ NH1] | 2.92 | a:GLU 403[ OE1] | e:ARG 405[ NH1] | 2.91 | d:GLU 403[ OE1] | e:LYS 387[ NZ ] | 3.76 | a:GLU 426[ OE1] |
| d:ARG 405[ NH1] | 3.57 | a:GLU 403[ OE2] | e:ARG 405[ NH1] | 3.45 | d:GLU 403[ OE2] | e:LYS 387[ NZ ] | 2.94 | a:GLU 426[ OE2] |
| d:ARG 405[ NH2] | 3.76 | a:GLU 403[ OE1] | e:ARG 405[ NH2] | 3.69 | d:GLU 403[ OE1] | e:ARG 435[ NH1] | 2.81 | a:ASP 438[ OD2] |
| d:ARG 405[ NH2] | 2.90 | a:GLU 403[ OE2] | e:ARG 405[ NH2] | 2.65 | d:GLU 403[ OE2] | e:GLU 403[ OE1] | 2.89 | a:ARG 405[ NH1] |
| d:LYS 412[ NZ ] | 2.67 | a:ASP 414[ OD1] | e:LYS 412[ NZ ] | 2.73 | d:ASP 414[ OD1] | e:GLU 403[ OE2] | 3.78 | a:ARG 405[ NH1] |
| d:LYS 412[ NZ ] | 3.23 | a:ASP 414[ OD2] | e:LYS 412[ NZ ] | 3.25 | d:ASP 414[ OD2] | e:GLU 403[ OE1] | 3.48 | a:ARG 405[ NH2] |
| d:GLU 426[ OE2] | 3.56 | a:LYS 387[ NZ ] | e:GLU 426[ OE1] | 3.52 | d:LYS 387[ NZ ] | e:GLU 403[ OE2] | 2.84 | a:ARG 405[ NH2] |
| d:ASP 438[ OD2] | 2.76 | a:ARG 435[ NH1] | e:GLU 426[ OE2] | 3.82 | d:LYS 387[ NZ ] | e:ASP 414[ OD1] | 2.85 | a:LYS 412[ NZ ] |
|  |  |  | e:ASP 438[ OD2] | 2.84 | d:ARG 435[ NH2] | e:ASP 414[ OD2] | 3.43 | a:LYS 412[ NZ ] |
|  |  |  | e:ASP 438[ OD2] | 3.81 | d:ARG 435[ NH1] |  |  |  |

**Supplementary Table 10: Comparison between three interfaces in A/Denver/57 hemagglutinin**

| **PDB ID: 6ML8**  **Number of H-bonds: 17**  **Number of Salt bonds: 11**  **Interface area [Å2]: 1519.3** | | | **PDB ID: 6ML8**  **Number of H-bonds: 17**  **Number of Salt bonds: 11**  **Interface area [Å2]: 1519.3** | | | **PDB ID: 6ML8**  **Number of H-bonds: 17**  **Number of Salt bonds: 11**  **Interface area [Å2]: 1519.3** | | |
| --- | --- | --- | --- | --- | --- | --- | --- | --- |
| **Protomer 1** | [**Dist. [Å]**](javascript:openWindow('pi_ipage_atmdist.html',400,250);) | **Protomer 2** | **Protomer 3** | [**Dist. [Å]**](javascript:openWindow('pi_ipage_atmdist.html',400,250);) | **Protomer 1** | **Protomer 3** | [**Dist. [Å]**](javascript:openWindow('pi_ipage_atmdist.html',400,250);) | **Protomer 2** |
| G:ARG 419[ NH1] | 2.69 | O:GLU 417[ OE1] | G:ARG 419[ NH1] | 2.69 | O:GLU 417[ OE1] | G:ARG 419[ NH1] | 2.69 | O:GLU 417[ OE1] |
| G:ARG 419[ NH1] | 2.83 | O:GLU 412[ O  ] | G:ARG 419[ NH1] | 2.83 | O:GLU 412[ O  ] | G:ARG 419[ NH1] | 2.83 | O:GLU 412[ O  ] |
| G:ARG 419[ NH2] | 2.90 | O:GLU 417[ OE2] | G:ARG 419[ NH2] | 2.90 | O:GLU 417[ OE2] | G:ARG 419[ NH2] | 2.90 | O:GLU 417[ OE2] |
| G:LYS 426[ NZ ] | 2.81 | O:ASN 424[ OD1] | G:LYS 426[ NZ ] | 2.81 | O:ASN 424[ OD1] | G:LYS 426[ NZ ] | 2.81 | O:ASN 424[ OD1] |
| G:LYS 426[ NZ ] | 2.92 | O:ASP 428[ OD1] | G:LYS 426[ NZ ] | 2.92 | O:ASP 428[ OD1] | G:LYS 426[ NZ ] | 2.92 | O:ASP 428[ OD1] |
| G:TYR 437[ OH ] | 3.07 | O:VAL 398[ O  ] | G:TYR 437[ OH ] | 3.07 | O:VAL 398[ O  ] | G:TYR 437[ OH ] | 3.07 | O:VAL 398[ O  ] |
| G:GLU 440[ OE2] | 2.57 | O:LYS 401[ NZ ] | G:GLU 440[ OE2] | 2.57 | O:LYS 401[ NZ ] | G:GLU 440[ OE2] | 2.57 | O:LYS 401[ NZ ] |
| G:ASP 452[ OD2] | 3.62 | O:ARG 449[ NH1] | G:ASP 452[ OD2] | 3.62 | O:ARG 449[ NH1] | G:ASP 452[ OD2] | 3.62 | O:ARG 449[ NH1] |
| G:ASP 452[ OD2] | 3.60 | O:ARG 449[ NH2] | G:ASP 452[ OD2] | 3.60 | O:ARG 449[ NH2] | G:ASP 452[ OD2] | 3.60 | O:ARG 449[ NH2] |
| A:GLN 121[ NE2] | 3.18 | O:LYS 415[ O  ] | A:GLN 121[ NE2] | 3.18 | O:LYS 415[ O  ] | A:GLN 121[ NE2] | 3.18 | O:LYS 415[ O  ] |
| A:LYS 321[ NZ ] | 2.60 | O:ASP 433[ OD2] | A:LYS 321[ NZ ] | 2.60 | O:ASP 433[ OD2] | A:LYS 321[ NZ ] | 2.60 | O:ASP 433[ OD2] |
| A:ASN 221[ O  ] | 3.60 | O:LYS 415[ NZ ] | A:ASN 221[ O  ] | 3.60 | O:LYS 415[ NZ ] | A:ASN 221[ O  ] | 3.60 | O:LYS 415[ NZ ] |
| A:GLU 117[ OE2] | 2.97 | O:LYS 418[ N  ] | A:GLU 117[ OE2] | 2.97 | O:LYS 418[ N  ] | A:GLU 117[ OE2] | 2.97 | O:LYS 418[ N  ] |
| A:GLU 117[ OE1] | 3.06 | O:LYS 418[ N  ] | A:GLU 117[ OE1] | 3.06 | O:LYS 418[ N  ] | A:GLU 117[ OE1] | 3.06 | O:LYS 418[ N  ] |
| A:GLU 117[ OE2] | 2.92 | O:ARG 419[ N  ] | A:GLU 117[ OE2] | 2.92 | O:ARG 419[ N  ] | A:GLU 117[ OE2] | 2.92 | O:ARG 419[ N  ] |
| A:GLU 117[ OE2] | 3.31 | O:ARG 419[ NE ] | A:GLU 117[ OE2] | 3.31 | O:ARG 419[ NE ] | A:GLU 117[ OE2] | 3.31 | O:ARG 419[ NE ] |
| A:GLU 120[ OE2] | 3.35 | O:ASN 422[ ND2] | A:GLU 120[ OE2] | 3.35 | O:ASN 422[ ND2] | A:GLU 120[ OE2] | 3.35 | O:ASN 422[ ND2] |
| **Salt Bridges** | | | **Salt Bridges** | | | **Salt Bridges** | | |
| G:ARG 419[ NH1] | 3.69 | O:GLU 417[ OE2] | G:ARG 419[ NH1] | 3.69 | O:GLU 417[ OE2] | G:ARG 419[ NH1] | 3.69 | O:GLU 417[ OE2] |
| G:ARG 419[ NH1] | 2.69 | O:GLU 417[ OE1] | G:ARG 419[ NH1] | 2.69 | O:GLU 417[ OE1] | G:ARG 419[ NH1] | 2.69 | O:GLU 417[ OE1] |
| G:ARG 419[ NH2] | 2.90 | O:GLU 417[ OE2] | G:ARG 419[ NH2] | 2.90 | O:GLU 417[ OE2] | G:ARG 419[ NH2] | 2.90 | O:GLU 417[ OE2] |
| G:ARG 419[ NH2] | 3.46 | O:GLU 417[ OE1] | G:ARG 419[ NH2] | 3.46 | O:GLU 417[ OE1] | G:ARG 419[ NH2] | 3.46 | O:GLU 417[ OE1] |
| G:LYS 426[ NZ ] | 2.92 | O:ASP 428[ OD1] | G:LYS 426[ NZ ] | 2.92 | O:ASP 428[ OD1] | G:LYS 426[ NZ ] | 2.92 | O:ASP 428[ OD1] |
| G:LYS 426[ NZ ] | 3.61 | O:ASP 428[ OD2] | G:LYS 426[ NZ ] | 3.61 | O:ASP 428[ OD2] | G:LYS 426[ NZ ] | 3.61 | O:ASP 428[ OD2] |
| G:GLU 440[ OE2] | 2.57 | O:LYS 401[ NZ ] | G:GLU 440[ OE2] | 2.57 | O:LYS 401[ NZ ] | G:GLU 440[ OE2] | 2.57 | O:LYS 401[ NZ ] |
| G:ASP 452[ OD2] | 3.62 | O:ARG 449[ NH1] | G:ASP 452[ OD2] | 3.62 | O:ARG 449[ NH1] | G:ASP 452[ OD2] | 3.62 | O:ARG 449[ NH1] |
| G:ASP 452[ OD2] | 3.60 | O:ARG 449[ NH2] | G:ASP 452[ OD2] | 3.60 | O:ARG 449[ NH2] | G:ASP 452[ OD2] | 3.60 | O:ARG 449[ NH2] |
| A:LYS 321[ NZ ] | 2.60 | O:ASP 433[ OD2] | A:LYS 321[ NZ ] | 2.60 | O:ASP 433[ OD2] | A:LYS 321[ NZ ] | 2.60 | O:ASP 433[ OD2] |
| A:GLU 117[ OE2] | 3.31 | O:ARG 419[ NE ] | A:GLU 117[ OE2] | 3.31 | O:ARG 419[ NE ] | A:GLU 117[ OE2] | 3.31 | O:ARG 419[ NE ] |

**Supplementary Table11: Comparison of interfaces between hemagglutinin and C05 antibody in A/Denver/57 strain**

| **PDB ID: 6ML8**  **Number of H-bonds: 8**  **Number of Salt bonds: 1**  **Interface area [Å2]: 786.1** | | | **PDB ID: 6ML8**  **Number of H-bonds: 4**  **Number of Salt bonds: 3**  **Interface area [Å2]: 397.8** | | | **PDB ID: 6ML8**  **Number of H-bonds: 12**  **Number of Salt bonds: 2**  **Interface area [Å2]: 1519.3** | | |
| --- | --- | --- | --- | --- | --- | --- | --- | --- |
| **Ab Heavy chain** | [**Dist. [Å]**](javascript:openWindow('pi_ipage_atmdist.html',400,250);) | **RBD** | **Ab Light chain** | [**Dist. [Å]**](javascript:openWindow('pi_ipage_atmdist.html',400,250);) | **RBD** | **Ab Light chain** | [**Dist. [Å]**](javascript:openWindow('pi_ipage_atmdist.html',400,250);) | **Ab Heavy chain** |
| H:SER 100C[ OG ] | 2.72 | A:GLU 203[ OE2] | L:THR  20[ N  ] | 3.22 | A:LEU  88[ O  ] | L:ARG  56[ NH1] | 3.62 | H:GLU   1[ OE1] |
| H:GLU 100G[ N  ] | 2.71 | A:VAL 148[ O  ] | L:ARG  18[ N  ] | 3.14 | A:ASN  90[ OD1] | L:GLN  38[ NE2] | 3.20 | H:GLN  39[ OE1] |
| H:ARG 100H[ NH1] | 3.28 | A:THR 144[ OG1] | L:ARG  18[ NH2] | 2.65 | A:GLU 129[ OE1] | L:TYR  87[ OH ] | 3.64 | H:LYS  43[ O  ] |
| H:ALA 100I[ N  ] | 2.67 | A:ARG 146[ O  ] | L:ARG  18[ O  ] | 3.35 | A:ASN  90[ N  ] | L:GLN  38[ NE2] | 3.81 | H:TYR  91[ OH ] |
| H:SER 100C[ O  ] | 3.81 | A:SER 240[ OG ] |  |  |  | L:GLN 160[ NE2] | 3.24 | H:LEU 170[ O  ] |
| H:ALA 100D[ O  ] | 2.79 | A:TYR 108[ OH ] |  |  |  | L:GLN  38[ OE1] | 3.07 | H:GLN  39[ NE2] |
| H:GLU 100G[ O  ] | 2.79 | A:VAL 148[ N  ] |  |  |  | L:TYR  87[ OH ] | 3.81 | H:GLN  39[ NE2] |
| H:GLU 100G[ OE1] | 3.52 | A:SER 158[ OG ] |  |  |  | L:LYS  42[ O  ] | 3.54 | H:TYR  91[ OH ] |
|  |  |  |  |  |  | L:TYR  36[ OH ] | 2.76 | H:PHE 100P[ N  ] |
|  |  |  |  |  |  | L:THR 129[ OG1] | 2.92 | H:LYS 143[ NZ ] |
|  |  |  |  |  |  | L:ASN 138[ OD1] | 2.51 | H:HIS 164[ NE2] |
|  |  |  |  |  |  | L:GLU 123[ OE1] | 3.58 | H:LYS 209[ NZ ] |
| **Salt Bridges** | | | **Salt Bridges** | | | **Salt Bridges** | | |
| H:ASP 100J[ OD1] | 3.84 | A:ARG 146[ NE ] | L:ARG  18[ NE ] | 3.75 | A:GLU 129[ OE1] | L:ARG  56[ NH1] | 3.62 | H:GLU   1[ OE1] |
|  |  |  | L:ARG  18[ NH2] | 2.65 | A:GLU 129[ OE1] | L:GLU 123[ OE1] | 3.58 | H:LYS 209[ NZ ] |
|  |  |  | L:ARG  18[ NH2] | 3.56 | A:GLU 129[ OE2] |  |  |  |
